# Learning Adsorption Patterns on Amorphous Surfaces

**DOI:** 10.1021/acs.jctc.4c00702

**Published:** 2024-08-26

**Authors:** Mattia Turchi, Sandra Galmarini, Ivan Lunati

**Affiliations:** † Laboratory for Computational Engineering, Swiss Federal Laboratories for Materials Science and Technology, Empa, Überlandstrasse 129, 8600 Dübendorf, Switzerland; ‡ Laboratory for Building Energy Materials and Components, Swiss Federal Laboratories for Materials Science and Technology, Empa, Überlandstrasse 129, 8600 Dübendorf, Switzerland

## Abstract

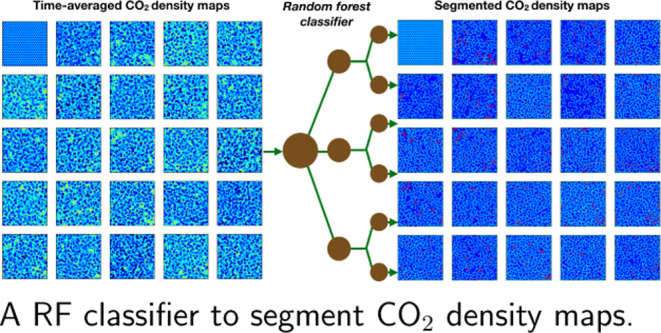

The physicochemical heterogeneity found on amorphous surfaces leads to a complex interaction of adsorbate molecules with topological and undercoordinated defects, which enhance the adsorption capacity and can participate in catalytic reactions. The identification and analysis of the adsorption structure observed on amorphous surfaces require novel tools that allow the segmentation of the surfaces into complex-shaped regions that contrast with the periodic patterns found on crystalline surfaces. We propose a Random Forest (RF) classifier that segments the surface into regions that can then be further analyzed and classified to reveal the dynamics of the interaction with the adsorbate. The RF segmentation is applied to the surface density map of the adsorbed molecules and employs multiple features (intensity, gradient, and the eigenvalues of the Hessian matrix) which are nonlocal and allow a better identification of the adsorption structures. The segmentation depends on a set of parameters that specify the training set and can be tailored to serve the specific purpose of the segmentation. Here, we consider an example in which we aim to separate highly heterogeneous regions from weakly heterogeneous regions. We demonstrate that the RF segmentation is able to separate the surface into a fully connected weakly heterogeneous region (whose behavior is somehow similar to crystalline surfaces and has an exponential distribution of the residence time) and a very heterogeneous region characterized by a complex residence-time distribution, which is generated by the undercoordinated defects and is responsible for the peculiar characteristics of the amorphous surface.

## Introduction

The surface of amorphous materials is characterized by topological disorder, which makes them very different from flat crystalline surfaces. In particular, amorphous silica surfaces are rough and populated by strained bonds typical of short-membered rings. In the presence of air moisture, the majority of the undercoordinated atoms at the surfaces react with water molecules and form hydroxyl groups (silanols). However, undercoordination defects such as nonbridging oxygen (NBO) and 3-fold coordinated silicon (Si3) can persist in fractions that depend on the specific manufacturing process. These defects were observed both experimentally^1−3^ and in numerical simulations by means of *ab initio* calculations^4−7^ and molecular dynamics (MD).^8−16^ These coordination defects can be active sites in catalytic reactions, such as methanation,^17,18^ and have a major impact on the adsorption of CO_2_.^10,19^ The physicochemical heterogeneity of amorphous surfaces is reflected in the irregular adsorption patterns that characterize the time-averaged density distribution of the molecules adsorbed on the surface (hereafter referred to as “density map”).

In our recent work, we compared the adsorption of CO_2_ onto crystalline and amorphous silica surfaces by means of MD simulations.^19^ The CO_2_ adsorption landscapes displayed some common patterns and key differences between crystalline and amorphous surfaces. On crystalline surfaces, the CO_2_ density map displays an ordered connected network, characterized by a periodic honeycomb structure. On amorphous surfaces, the connected network is distorted and interspersed by higher-density regions. These irregular adsorption patterns require new tools that are able to capture the topology of the adsorption landscape and describe the adsorption capacities of the different regions. A straightforward approach to define and classify surface features is to perform a segmentation of the density maps. Image segmentation consists in the classification of the image pixels based on some pixels attributes (features) and in the subsequent partition of the image into regions of connected pixels (segments).^20−22^

There are two classes of algorithms for pixels classification: supervised algorithms, which use labeled training data, and unsupervised algorithms, which do not. In general, supervised algorithms deliver more accurate predictions, conditional to the fact that the algorithm is trained with reliable labeled data.^23^ Among supervised methods, the Random Forest (RF) algorithm^24^ has been extensively used in different image-analysis applications, ranging from tissue identification in medical images^25^ to land classification in remote satellite images.^26^ Belonging to the family of tree-based algorithms, RF uses different trees to probabilistically classify pixels by majority votes.^24^ Each tree is built by randomly selecting a subset of the training data and of the pixels features. By introducing randomness into tree construction, predictions performed by each tree are less correlated and the overall prediction is less prone to overfitting.^24,27^

Here, we propose employing a Random Forest (RF) algorithm for pixel classification and segmentation of the two-dimensional (2D) density maps that are generated by MD simulations of silica/CO_2_ interfaces. Our objectives are (i) to develop a robust and flexible strategy to select reliable labeled data (which are used to train the RF classifier) based on detailed analysis of pixels features, (ii) to employ the trained classifier to segment the time-averaged surface density maps generated by means of MD simulations, and (iii) to assess the quality of the segmentation based on the uncertainty of classification as well as on application inspired criteria.

## Methods

The RF segmentation is applied to density maps obtained from MD simulations of CO_2_ adsorption on amorphous and crystalline surfaces. After generation of the crystalline samples, the amorphous pores are obtained by means of well-established melt and quench protocols. Then, MD simulations of the CO_2_/silica interfaces are performed to produce the CO_2_ density maps at the pore surface. The quality of the segmentation is assessed by considering the uncertainty of the classification as well as specific properties, calculated for the classified regions, which can be important to characterize the surface or to build larger-scale models.

### Generation of the Silica Surfaces

We consider one hydroxylated crystalline pore and three classes of hydroxylated amorphous pores. The surfaces of the crystalline pore are generated from the cleavage of a β-cristobalite supercell along the 111 crystallographic face and are characterized by a hydroxyl surface density of 4.5 OH/nm^2^.^28,29^ The slit nanopores are formed by two slabs which extend for around 100 Å × 100 Å × 38 Å in *x*, *y*, and *z* directions, respectively. The width of the pores (in the *z* direction) is 2 nm. Periodic boundary conditions are applied in all three directions. This pore size has been used both for amorphous^30^ and crystalline silica pores interacting with CO_2_.^31^ We remark that molecules are not affected by the silica surfaces if they are at a distance larger than 0.5 nm and that the concentration of the CO_2_ molecules reaches a constant bulk value in the center of the pore (see Figures S1 and S2 in the Supporting Information).

To take into account the statistical variability of the amorphous pores we generate four samples for each of the three classes. Melt and quench is the most common procedure to generate amorphous materials by means of MD simulations.^14,15,32,33^ Here, we follow the protocol proposed by Du et al. which uses the Buckingham potential^30,34^ (see Turchi et al.^19^ for the detailed description of the generation of the amorphous surfaces).

The three classes of amorphous pores differ by the temperature selected to anneal the surface layers, *T*_a_, which determines the number and the type of surface sites (namely, hydroxyl groups, OH; undercoordinated or nonbridging oxygen, NBO; and undercoordinated silicon, Si3). Different annealing temperatures generate different degrees of amorphism and, most importantly, different surface densities of Si3, NBO, and OH. We refer to the classes generated by different annealing temperatures as (1) LowAm (*T*_a_ = 2000 K) with the lowest density of surface sites, (2) MedAm (*T*_a_ = 1500 K) with the intermediate density (ID) of surface sites, and HighAm (*T*_a_ = 1000 K) with the highest density of surface sites. Once generated, the amorphous surfaces are hydroxylated by means of an in-house code which follows the protocol^16,19,30^ to hydroxylate (respectively, hydrogenate) all exposed Si3 (respectively, NBO) sites which result from the melt and quench simulation, as well as the 2 and 3-membered rings (identified according to the Guttman^35^ definition) which are broken to allow for the grafting of the OH groups. After hydroxylation, the surfaces are equilibrated for 1 ns in the NVT ensemble using the ClayFF potential,^36^ which is later employed to simulate the CO_2_/silica interface.

Some of the undercoordinated defects cannot be functionalized due to the surface roughness which renders some sites inaccessible to water molecules and prevents hydrogenation or hydroxylation.^19^ The final surface densities of Si3, NBO, and OH groups are reported in the Supporting Information (Table S1). Note that the hydroxyl surface density is in the range of the experimental values reported by Zhuravlev et al.^37^ and the concentration of surface defects (i.e., Si3 and NBO) agrees with those obtained by reactive MD simulations^9^ or measured by infrared spectroscopy.^2,38^

All amorphous surfaces display roughness in the range between 0.5 and 0.6 nm regardless of the annealing temperature (see ref (19) for the estimation of the surface roughness). Roughness in this range was reported for amorphous silica/water interface characterized by ultrathin tips atomic force microscope measurements^39^ and for ultrasmooth silica surfaces.^40^ If necessary, the level of roughness can be conveniently tuned via MD to match other experimental values.^33,41^ For instance, an approach to randomly generate rough cleaving planes characterized by different degrees of roughness (i.e., in the range between 0.6 and 2.3 nm), was recently proposed by Nguyen et al.^42^ Notice, however, that our goal is to devise a methodology to investigate and characterize the irregularly shaped structures arising on amorphous surfaces, and the slit pores generated by the melt-and-quench protocol are sufficiently challenging in this respect.

### Molecular Dynamics Simulations and CO_2_ Density Map

The dynamics of 100 CO_2_ is simulated in the 13 silica slit nanopores (1 crystalline pore and 12 amorphous pores, 4 for each class) by means of Molecular Dynamic (MD) (performed with LAMMPS^43^). Populating a pore volume of 200 nm^3^ with 100 CO_2_ molecules leads to a density of around 36 kg/m^3^ and a partial pressure of around 19 atm (at the simulation temperature, *T* = 290 K, this corresponds to a CO_2_ gas phase). This number of molecules, (which was used also in previous studies^19,31^) has been chosen as it offers a good compromise between effective sampling of the surface and minimization of the CO_2_–CO_2_ interactions.^19^

ClayFF^36^ is employed to describe the hydroxylated silica and the dynamics and surface bonding of the CO_2_ molecules, which are described by a flexible version of the EPM2 model.^44,45^ For the SiO_2_/CO_2_ cross interactions terms we employ the parameters optimized and validated by Crabtree et al.^46^ and Purton et al.^47^ All parameters used in the ClayFF and EPM2 FF can be found in Turchi et al.^19^ The reliability of ClayFF to treat amorphous silica has been demonstrated in several works. Bourg et al.^48^ reported that ClayFF yields hydroxylated amorphous silica structures in agreement with experimental data at room temperature.^49,50^ Leroch et al.^51^ showed that ClayFF provides an accurate description of humid amorphous silica surfaces and yields water adsorption isotherms for hydroxylated amorphous silica pores which agree very well with experimental data.^52^

All simulations are performed in the NVT ensemble with a time step of 1 fs. Two separated Nosè–Hoover^53,54^ thermostats, with a relaxation time of 100 fs, keep the temperatures of the CO_2_ and the silica substrate at 290 K. Each simulation is run for 100 ns of which the last 90 ns are used for analyzing results. A cutoff of 10 Å is employed for the short-range van der Waals as well as for the electrostatic interactions. The long-range Coulombic interactions are treated with the staggered 3 particle–mesh (PPPM) method,^55−57^ with an accuracy of 10^–4^.

Density maps^58^ are generated by tracking the position of the carbon atoms of CO_2_ molecules adsorbed onto each pore surface. CO_2_ molecules are considered adsorbed if they are within a cutoff distance from the surfaces (see Figure S2 in the Supporting Information). The position is mapped onto a 2D histogram of 500 × 500 bins for a total of 90,000 frames from the last 90 ns of the simulation. For each of the 26 surfaces, the density map is obtained by averaging the density distribution of the adsorbed molecules over time.

### Segmentation of the CO_2_ Density Maps

**Figure 1:**
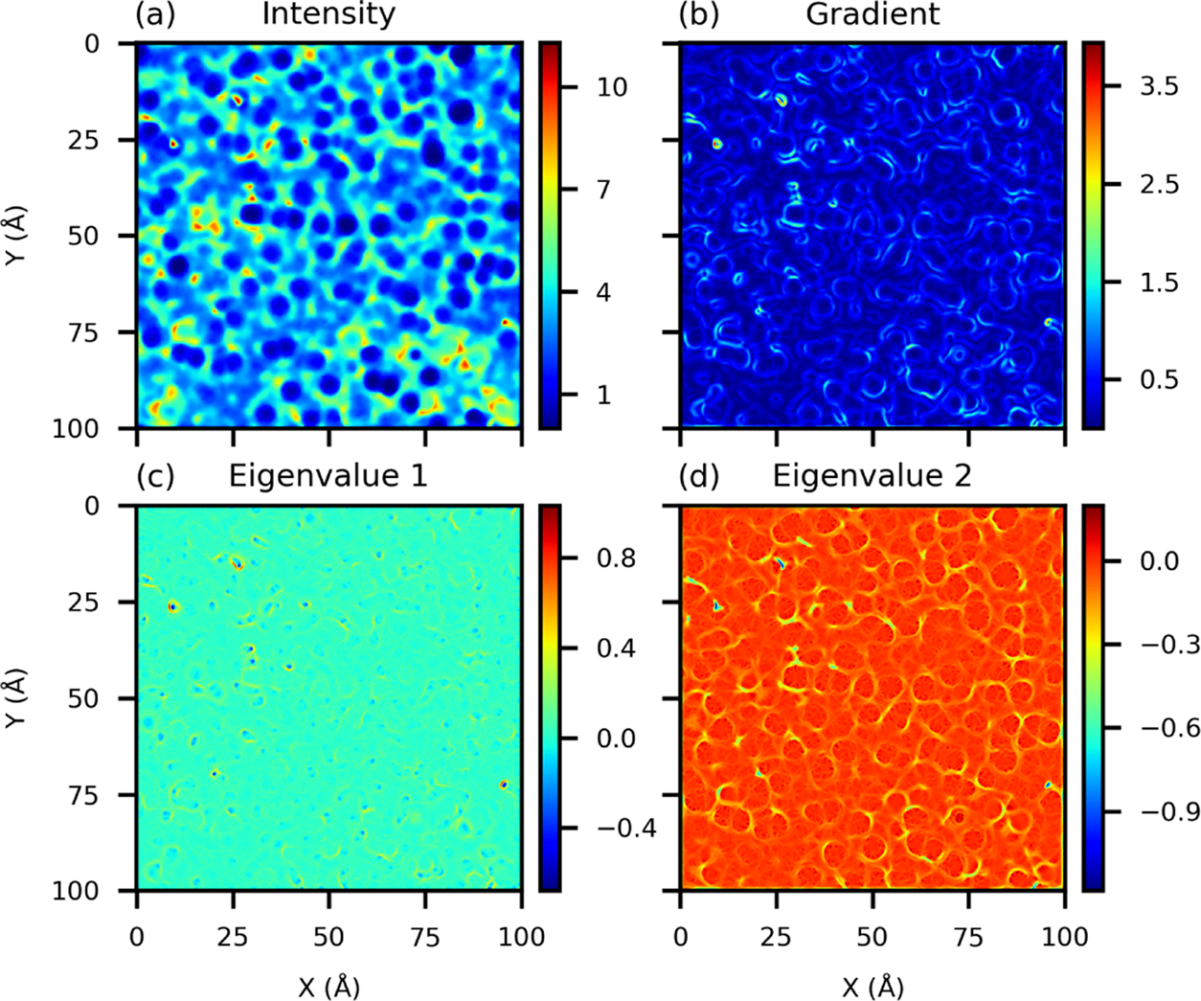
Different features characterizing the CO_2_ density maps: pixel intensity (a), absolute value of the gradient of the pixel intensity (b), first eigenvalue of the Hessian matrix (c), and second eigenvalue of the Hessian matrix (d).

Regions of different adsorptive capacities can be identified from the analysis of the density maps of crystalline and amorphous pore surfaces.^19^ The density maps of the crystalline surfaces are characterized by an ordered network of intermediate density (InterDens, ID) and well-defined hexagonal low density (LowDens, LD) in which CO_2_ does not adsorb (or adsorbs for a negligible amount of time). On the other hand, amorphous surfaces display high-density (HighDens, HD) regions embedded in a distorted network of intermediate density (InterDens). The rest of the domain is characterized by LowDens regions of irregular shapes (Figure 1).

Our hypothesis,^19^ is that the LowDens and InterDens regions at the crystalline and amorphous surfaces have comparable adsorption capacities, as they both result from hydroxyl groups at the surface. On the other hand, the HighDens at the amorphous surfaces is the very signature of the disordered structure of the amorphous surfaces, which produces undercoordinated defects at the surface, in addition to the distorted network of intermediate density.

Therefore, the (three-class) segmentation of the density maps of the amorphous surfaces is performed to identify the HighDens regions resulting from surface defects and separate them from the InterDens and LowDens regions. The HighDens regions can then be separately characterized, whereas InterDens and LowDens regions can be compared with those obtained from the (two-class) segmentation of the density maps of the crystalline surfaces, which identify LowDens and InterDens regions.

#### Preprocessing of the Density Maps

To enhance the contrast between the different classes (which are hidden by the presence of HighDens regions that display values that are up to 100 times larger than those of the InterDens regions), a logarithmic transformation is first applied to the density maps. Then, background noise is removed by means of the total variation algorithm introduced by Chambolle et al.^59^ and by applying a Gaussian smoother with kernel standard deviation σ = 1 (the applied filters are reported in Figure S3 in the Supporting Information).

#### Pixel Classification and Segmentation of the Density Maps

To classify each image pixel and perform a segmentation of the density maps, we employ Random Forest (RF), a tree-based classification algorithm. RF consists of multiple decision trees, which are independent supervised learning algorithm trained on randomly selected subsamples of the data. Averaging the classification response of uncorrelated trees reduces the overall variance and prediction error, hence the risk of overfitting.^24^

The tree has a hierarchical structure consisting of root nodes, branches, internal nodes, and terminal nodes. Data are input into the root node and are partitioned into further branches that feed the internal nodes based on data features and specified criteria, which measures the best features split (e.g., Gini impurity). The process is repeated at each internal node for an increasingly smaller portion of data until the terminal nodes output the class to which each data is attributed. At each node partition (split), another instance of randomness is introduced by randomly drawing a subset of features, which is used to make the tree prediction. The final classification is assigned according to the majority of votes, i.e., the predictions of the trees in the forest.^27^

The methodology consists of the following main steps:

- Definition of the features and random sampling of training data. The data to train the RF algorithm are sampled from the pixels (data) of conveniently defined subdomains of the postprocessed images (the density maps). Four features are considered: the pixel intensity, *I*; the absolute value of the intensity gradient, *G*; and the two eigenvalues of the Hessian matrix, *E*^1^ and *E*^2^. Based on the sign of *E*^1^ and *E*^2^ and on values of *I* and *G*, we define three distinct subdomains, *D*_*j*_ with *j* ∈ LD, ID, HD, which are assumed to belong to three adsorption regions/classes. The sampling subdomains are defined by appropriate cutoff values applied to *E*^1^, *E*^2^, *G*, and *I*. As varying the cutoffs results in different segmentation, we test and evaluate the performance of different cutoff values. We randomly select the data to train the trees from these subdomains and we obtain the training data set *d*_*j*,*i*_, where *i* = 1, 2, ···, *m*_*j*_ indicates the index of the *m*_*j*_ pixel attributed to the *j*-class in the subdomain *D*_*j*_.
  Note that the use of features that depends on the derivatives of the intensity up to the second order allows the RF to classify the pixel of the density maps on the basis not only of pixel intensity (as done, e.g., by a simple thresholding algorithm), but taking into account also the information about the neighboring pixels, hence capturing the nonlocal features of the density maps.
- Image smoothing. To reduce the impact of inhomogeneity of the intensity fields, as well as the noise in the gradients and eigenvalues of the Hessian matrix,^60^ 4 levels of Gaussian smoothing (σ = 1, 2, 4, 8) are applied to the density maps, and the RF uses all 4 features calculated for each smoothing level (hence, a total of 16 features). Smoothing is essential to detect objects with weak (not prominent) boundaries, such as blood vessels in medical images.^60,61^
- Selection of the hyperparameters. Once the training sets and the relevant features have been specified, we need to select the RF hyperparameters, which are not learned during the training but have to be specified beforehand. In the RF algorithm, the most important hyperparameters are the number of trees in the forest, *M*, and the number of drawn features at each split (more detailed information about the RF hyperparameters can be found in the literature^24,62^). As the pixel is classified based on majority vote of trees, increasing *M* generally leads to less uncertain classifications and should be sufficiently large.^27,62,63^ Here, we selected *M* = 1000 and mtry = 4 (i.e., square root of the total number of features, 16) and the Gini impurity criterion for the best features split.^64^
- Pixel classification and maps segmentation. Based on the majority votes, RF provides the probability that a pixel belongs to the *j*-class (LowDens, MedDens, or HighDens), *p*_*j*_ = *M*_*j*_/*M*, where *M*_*j*_ is the number of trees classifying the pixel as belonging to the *j*-class. Hence, each pixel is associated with a probability vector ***p*** = [*p*_LD_, *p*_ID_, *p*_HD_]. Finally, maps are segmented by connecting all of the adjacent pixels that belong to the same class, resulting in *n*_LD_, *n*_ID_, and *n*_HD_ objects attributed to the class LowDens, InterDens, and HighDens, respectively (*n* = *n*_LD_ + *n*_ID_ + *n*_HD_); each object is characterized by an area *A*_*j*,*k*_ where *j* ∈ LD, ID, HD and *k* = 1, 2, ···, *n*_*j*_.

### Target Properties in the Segmented Regions

To assess the quality of the segmentation, we consider, in addition to uncertainty measures, specific target properties that are estimated for the segmented regions. These properties should be defined according to the goals of the segmentation and the information that needs to be extracted from the MD simulations. Here, we focus on predicting the residence-time (or adsorption time) distribution and the spatial average densities of each segmented object. These quantities allow us to extract information about sorption dynamics that can be used, for instance, to build macroscale models. Also, they help define regions that deviate from the adsorption behavior of hydroxyl groups and are, therefore, related to the presence of undercoordinated defects. After identification, high-density regions can be correlated to the underlying defect distribution and to the specific atomic arrangement.

To define the adsorption time distribution, we consider an event as adsorption into the surface layer. To define the adsorbed molecules, we introduce a cutoff in the direction perpendicular to the pores surfaces (*z*): a molecule is adsorbed if its distance from the surface is smaller than the cutoff (Figure S2 in the Supporting Information). Hence, the number of events, *N*_ads_, is defined by the number of times a molecule enters the surface layer, whereas the event duration is the time interval elapsed before the molecule is released back into the pore. For each event, we then compute the fraction of time that is spent in each surface region, *n*_*j*,*k*_, defined by the 2D segmentation of the density map, which defines the boundaries of the segmented objects in the plane of the pore surface (hence, in the *x*, *y* directions). Therefore, the residence time in each segmented object is defined as the total time, *t*_*n*_*j*,*k*__, that the molecule spent in that object during an event.

We look at different initial time frames and track the adsorbed molecules in successive time windows of 400 ps (discretized into 400 time frames separated by 1 ps). To improve the statistics of the adsorption events, a total of 225 nonoverlapping time windows are considered over the final 90 ns of the simulation. The residence-time distribution of *n*_*j*,*k*_ is described by plotting at any time *t* the fraction of molecules that have total residence time *t*_*n*_*j*,*k*__ ≥ *t*. We assume that the residence time is described by a function of the form:
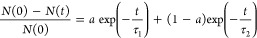
1

where *N*(*t*) is the number of molecules with total residence time *t*_*n*_*j*,*k*__ ≥ *t* (*N*(0) is the number of molecules adsorbed at time *t* = 0).

Equation 1 is a double exponential with a fraction *a*, respectively (1 – *a*), of the molecules characterized by a time constant τ_1_, respectively τ_2_. The same analysis is applied for the residence time in the surface layer (hence considering the event duration time). First, a fit to an exponential function (i.e., *a* = 1) is attempted. If the data are not satisfactorily described, they are fitted to a double-exponential function, and *a* ≠ 1 is determined together with τ_1_ and τ_2_ (eq 1). The error on the parameters is estimated as the standard deviation, which is calculated from the diagonal term in the covariance matrix. If the error on the second time constant is larger than its value, the data are still fitted to a single exponential. Note that the mean adsorption time (or mean lifetime of the molecule within the region of interest) is τ̅ = *a*τ_1_ + (1 – *a*)τ_2_.

For each segmented region, *n*_*j*,*k*_ we also compute the spatial average of the density,^58,65,66^
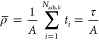
2

where *A* is the area of the segmented object, *t*_*i*_ is the residence time in the segmented object during the *i*th of the *N*_ads,*k*_ adsorption events (*N*_ads,*k*_ = ϕ_*k*_*N*_ads_, where ϕ_*k*_ takes into account that the *k* object is not visited during all layer adsorption events, *N*_ads_), and τ is the total residence time in the object. (Note that, to keep the notation simple, we have dropped the indexes *j* and *k* referring to the *j*th class and the *k*th segmented object, respectively. All quantities in eq 2 are defined for each *k*th segmented object belonging to the *j*-class, i.e., ρ̅ = ρ̅_*j*,*k*_, *A* = *A*_*j*,*k*_, *t*_*i*_ = *t*_*i*,*j*,*k*_, τ̅ = τ̅_*j*,*k*_; and in eq 1 for the *j*-class, i.e., *N*(*t*) = *N*_*j*_(*t*), *a* = *a*_*j*_, τ_1,*j*_, and τ_2,*j*_.

## Results

The results are organized into three parts. First, we investigate the effects of the subdomains from which the elements of the training sets for the RF algorithm are sampled on the quality of the segmentation. For each surface, we consider 16 different subdomains (defined by 16 different sets of parameters) and evaluate the segmented map by visual inspection and based on pixels uncertainty.

Based on this evaluation, we select 2 of the 16 set of parameters that lead to markedly different segmentation, and we characterize the resulting regions in terms of the properties of interest, namely, the residence time and spatial-averaged density of each region. Finally, we discuss the implications for crystalline and amorphous surfaces.

### Selection of the Sampling Subdomains and Effects on Segmentation

The training data, *d*_*j*,*i*_, are randomly sampled from three subdomains that can be (almost certainly) attributed to the class *j* based on the intensity of the density maps (*I*), the absolute value of its gradient (*G*), and the two eigenvalues (*E*^1^ and *E*^2^) of the Hessian matrix.

An example of these quantities is given in Figure 1. From the intensity map (Figure 1a), we note that LowDens and HighDens regions correspond to local minima and maxima, respectively, whereas the InterDens regions correspond to regions close to saddle points of the density map. Indeed, the regions that are characterized by positive values of the second eigenvalue (*E*^2^ > 0 in Figure 1d) are correlated with LowDens regions and are preselected for random sampling of *d*_LD,*i*_. Similarly, we use the first eigenvalue to preselect the regions to random sample the training data for InterDens (i.e., the saddle points, *E*^1^ > 0 and *E*^2^ < 0) and HighDens (the local maxima, *E*^1^ < 0 and *E*^2^ < 0). (As the crystalline surface is segmented only into two classes, regions are preselected only according to the second eigenvalue.)

These three subdomains are further restricted to reduce the risk of misclassification in the training set as well as to control the results of the segmentation and tailor it to the specific objectives and prediction of specific properties (i.e., residence time distribution and spatial-averaged density). Although the choice of the boundaries to be applied to the training data sampling subdomains is arbitrary, their rational selection can be guided by the inspection of Figure 1. First of all, regions characterized by a large absolute value of the intensity gradient are excluded from the training domain because they are typically at the boundary of the InterDens regions (Figure 1b) and their attribution to one of the three classes is uncertain.

Boundaries are set also on the other features and, in particular, on the second eigenvalue, which has a significant impact on target quantities (e.g., on the prediction of residence-time distributions within the segmented region, as it will be shown in the following sections). In summary, we define the subdomains from which the training data are randomly sampled as follows:

- local minima for *D*_LD_: *E*^2^ > 0, *E*^1^ > 0, *G* < *k*_G_, *I* < *k*_I,LD_;
- saddle points for *D*_ID_: *E*^2^ < −*k_E_*_^2^,ID_, *E*^1^ > 0, *G* < *k*_G_, *I* < *k*_I,ID_;
- local maxima for *D*_HD_: *E*^2^ < −*k_E_*_^2^,HD_, *E*^1^ < 0, *G* < *k*_G_, *I* > *k*_I,HD_,

where all of the constants (*k*) are positively defined.

**Figure 2:**
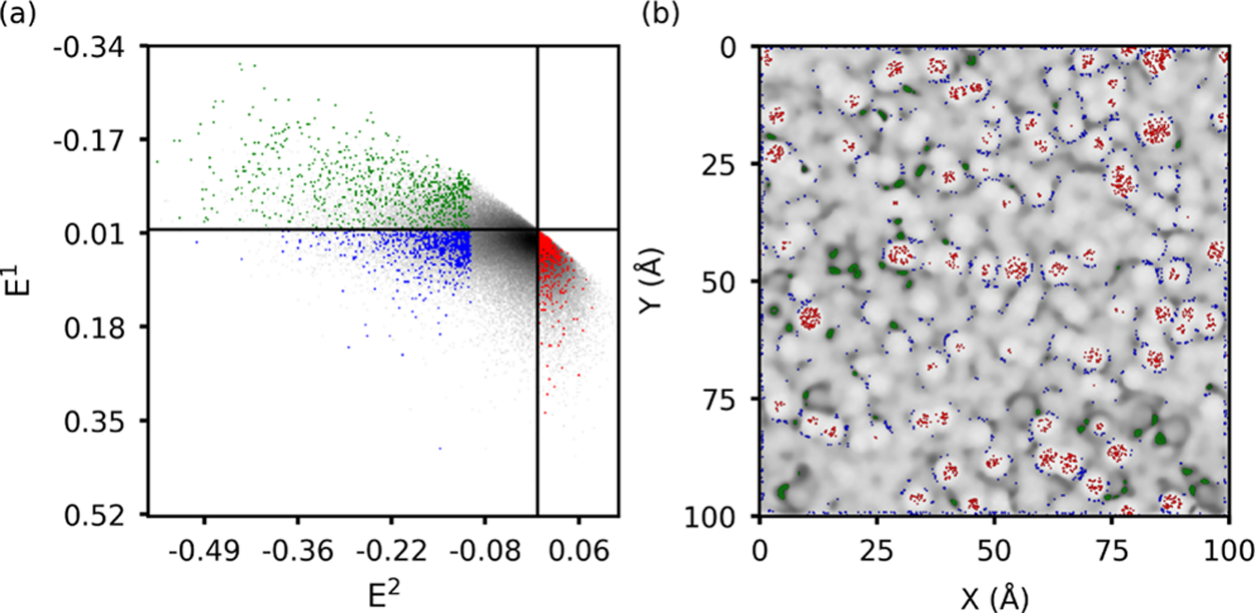
Pixels selected in the training of the RF algorithm for LowDens (red), InterDens (blue), and HighDens (green). (a) Pixels are displayed in the *E*^1^ – *E*^2^ space (the horizontal and vertical black lines separate the positive and negative values of *E*^1^ and *E*^2^, respectively). (b) The same pixels are depicted in the physical space of the intensity map (on a gray scale).

An example of the final sampling domains selected for the LowDens, InterDens, and HighDens regions is depicted in Figure 2 for one of the amorphous surfaces. Figure 2a shows how the constants reduce the subregions in the *E*^1^ – *E*^2^ space, from which the training data are randomly sampled for LowDens, InterDens, and HighDens, while the corresponding data sampled in the physical space are depicted in Figure 2b, together with the intensity map. Notice that unbalanced training data set (i.e., training set that do not contain the same number of data for each class) are likely to yield low classification accuracy of the underrepresented classes.^26^ To obtain a balanced training set, we randomly selected an equal number of data sets for the three classes. The class characterized by fewer data points limits the number of randomly selected pixels for the other two classes.

Based on the results of a preliminary sensitivity analysis, we set *k*_G_ equal to 1/5 of the highest value of *G* for all of the surfaces, as well as *k*_I,LD_ = 1, *k*_I,ID_ = 5 and *k*_I,HD_ = 7.5. The values of *k_E_*_^2^,ID_ and *k_E_*_^2^,HD_ are found to be the ones that mostly affect the segmentation results and are specifically selected for each surface by performing a grid search that explores all of the possible combinations of *k_E_*_^2^,ID_, *k_E_*_^2^,HD_ ∈ [−0.20, −0.15, −0.10, 0.0]. This yields a total of 16 combinations of the parameters resulting in 16 different segmentations of each density map (an example of all of the 16 segmentations for one surface is given in Figure S4 in the Supporting Information).

**Figure 3:**
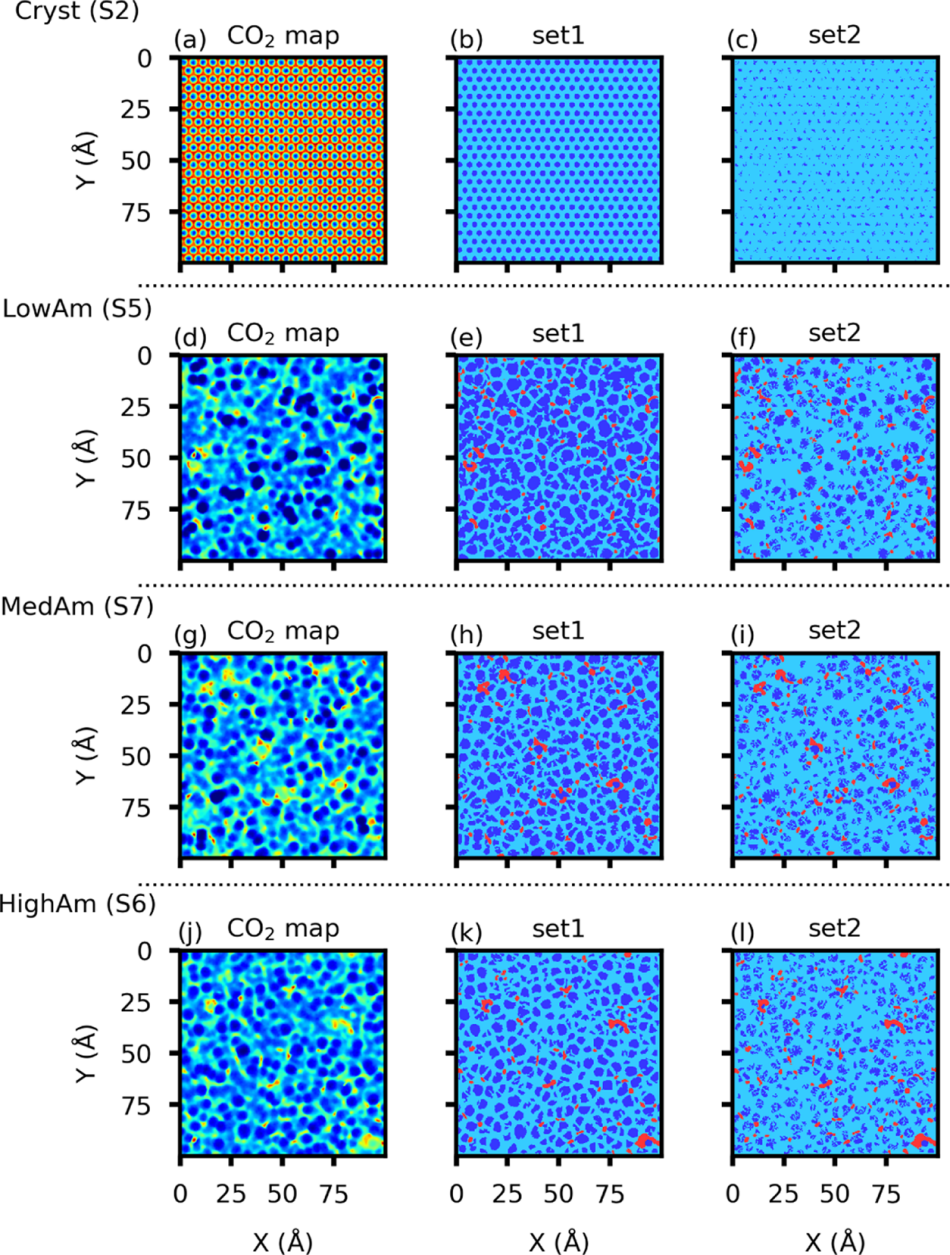
Time-averaged CO_2_ density maps (panels in the left column) and segmented maps performed with set1 (middle column) and set2 (right column) for reference samples of crystalline (a–c), LowAm (d–f), MedAm (g–i), and HighAm (j–l) surfaces. The reference sample surfaces for which the maps are shown are indicated in parentheses (i.e., S2 for Cryst). Segmented regions for the LowDens, InterDens, and HighDens classes are colored blue, light blue, and red, respectively.

By comparing the surface areas attributed to the three classes by different sets of segmentation parameters, we identify two types of segmentation results characterized by different patterns that depend on the choice of the constant *k_E_*_^2^,ID_ (see Figure 3 for an example):

- for *k_E_*_^2^,ID_ < 0 and *k_E_*_^2^,HD_ = ∈ {−0.20, −0.15, −0.10, 0.0} (set1), we obtain a similar total surface area for LowDens and InterDens, whereas
- for *k_E_*_^2^,ID_ = 0 and *k_E_*_^2^,HD_ = ∈ {−0.20, −0.15, −0.10, 0.0} (set2), InterDens has a considerably larger surface area than LowDens.

Both sets (i.e., set1 and set2) yield a fully connected network corresponding to a single InterDens region, whereas the LowDens and HighDens regions are separated into distinct objects. We consider both types of segmentation and for each of the two sets we select the combination of *k_E_*_^2^,ID_ and *k_E_*_^2^,HD_ which minimizes the median values of the Shannon entropy,
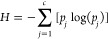
3The median values of the Shannon entropy (*H̃*) for each of the 16 segmentations of each surface are reported in the Supporting Information (Table S2).

### Comparison of Segmentation Uncertainty across Different Classes

**Figure 4:**
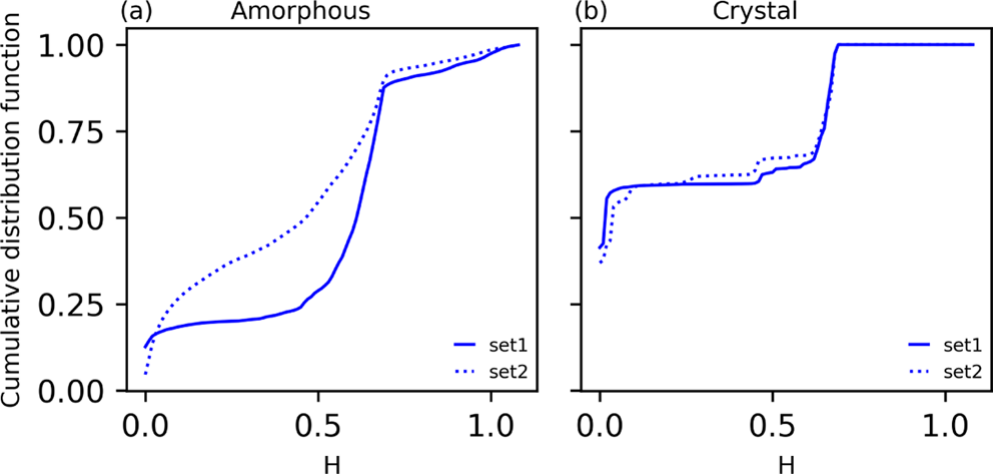
Cumulative distribution function of the Shannon entropy from which the median values of the *H* metric, *H̃*, is calculated. Curves are obtained for all 24 amorphous surfaces (a) and the 2 crystalline surfaces (b). Set1 yields *H̃* = 0.62 and 0.02 for the amorphous and crystalline surfaces, respectively. Set2 yields *H̃* = 0.47 for the amorphous surfaces and *H̃* = 0.05 for the crystalline surfaces.

The cumulative distribution functions of the Shannon entropy, *H*, obtained with set1 and set2 are shown in Figure 4 for the 24 amorphous surfaces and for the 2 crystalline surfaces. Higher areas under the curves correspond to a higher frequency of pixels with low Shannon entropy and, therefore, to a lower uncertainty in pixels classification. The profiles and the *H̃* values show that the segmentation obtained with set2 parameters is characterized by lower uncertainty for the amorphous surfaces, whereas the uncertainty of the two sets is comparable for the crystalline surface. Segmentation of crystalline surfaces is less uncertain than for the amorphous ones, which is expected since the regularly shaped honeycomb patterns of the InterDens are clearly separated from LowDens regions, Figure 3.

To understand how the uncertainty is distributed among each segmentation class for set1 and set2, we compute class-specific uncertainty metrics. As reported in Table 1 and depicted in Figure 3, sets 1 and 2 lead to different segmentation patterns and attribute a different number of pixels (hence, different surface areas) to the different classes. Set1 attributes a comparable number of pixels to LowDens and InterDens (above 45% for each class), whereas the pixels attributed to HighDens are limited to about 4%. Instead, set2 assigns a sensibly larger fraction of pixels to InterDens (around 65%) and to HighDens (around 7%), whereas fewer pixels are attributed to LowDens (around 26%).

**Table 1:** Mean Values (Averaged over All Surfaces of the Pore Class, i.e., S1–S8 for the Amorphous and S1–S2 for the Crystalline Surfaces) of the Areas and Median Values of the Shannon Entropy (*H̃*) Attributed to Each Segmentation Class (LowDens, MedDens, and HighDens) for the Two Sets of Parameters Used in the Segmentation (Standard Deviation Is Reported in Parentheses)^a^

		LowDens		InterDens		HighDens	
		set1	set2	set1	set2	set1	set2
*A* (nm^2^)	LowAm	48.5 (5.2)	25.0 (1.9)	48.0 (5.5)	67.4 (2.8)	3.5 (0.7)	7.6 (2.2)
	MedAm	46.9 (4.2)	26.0 (1.8)	49.1 (4.2)	67.3 (1.9)	4.0 (0.5)	6.8 (2.1)
	HighAm	47.5 (5.2)	27.3 (1.2)	49.1 (5.4)	64.9 (2.8)	3.4 (0.5)	7.9 (3.0)
	Cryst	28 (0.0)	4 (0.7)	72 (0.0)	96 (0.7)		
*H̃*	LowAm	0.64 (0.03)	0.65 (0.02)	0.56 (0.04)	0.32 (0.11)	0.55 (0.02)	0.57 (0.04)
	MedAm	0.66 (0.04)	0.66 (0.03)	0.59 (0.06)	0.26 (0.14)	0.52 (0.12)	0.56 (0.04)
	HighAm	0.63 (0.04)	0.65 (0.03)	0.47 (0.16)	0.37 (0.16)	0.43 (0.21)	0.59 (0.05)
	Cryst	0.63 (0.00)	0.69 (0.00)	0.02 (0.003)	0.06 (0.01)		

a Extended version of the table containing the data for each surface is reported in the Supporting Information (Table S3).

**Figure 5:**
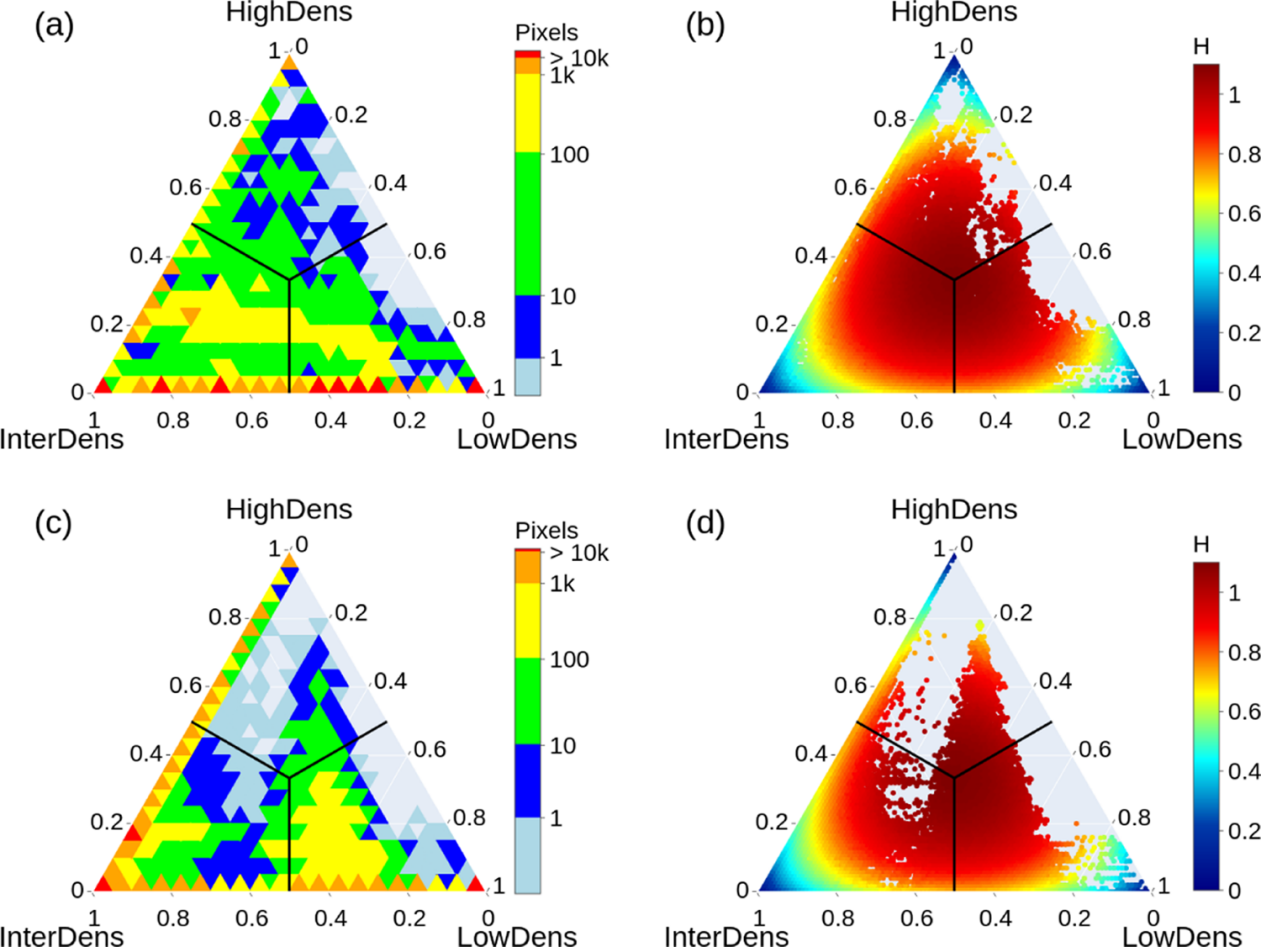
Ternary diagrams of the distribution of the probability vectors (left columns) and of the Shannon entropy of the classified pixels (right columns). The pixels are classified according to the highest components of the probability vector; the space regions attributed to the different classes (LowDens, InterDens, HighDens) are separated by the three thick black lines. The probability vectors are mapped into triangular bins of side 0.05, and the color scale corresponds to the number of pixels in each bin corresponding to set1 (a) and set2 (c). Note that all pixels relative to the 24 amorphous surfaces are considered, and the values are normalized by the number of surfaces. In panels (b) and (d), each pixel of the 24 amorphous surfaces is colored based on the relative value of the Shannon entropy (*H*) for set1 and set2, respectively. The Shannon entropy ranges from the minimum value *H* = 0 for pixels located at the vertices of the triangle to the maximum value for pixels located at the triple point (the intersection of the three black lines, *p*_LD_ = *p*_ID_ = *p*_HD_ = 1/3).

The distribution of the probability vectors, ***p***, associated with each pixel of the 24 amorphous surfaces across the LowDens, InterDens, and HighDens regions are displayed in Figure 5, together with the corresponding values of the Shannon entropy, *H*. In the ternary diagrams of Figure 5a,c, probability vector data are arranged into triangular bins (with discrete intervals of 0.05 in each *p*_*j*_ component for a total of 100 bins), and the number of pixels in each bin is normalized by the number of surfaces taken into consideration. The ternary diagrams of Figure 5b,d show the Shannon entropy of each pixel of the 24 surfaces. The minimum value (*H* = 0) is located at the vertices of the triangle and corresponds to zero uncertainty, whereas the maximum value is located at the triple point (*p*_LD_ = *p*_ID_ = *p*_HD_ = 1/3).

Both for set1 and set2, the majority of pixels are attributed either to bins located at the vertices and classified with very low uncertainty or on LowDens–InterDens and InterDens–HighDens axes (Figure 5). The histograms of pixels at the LowDens–InterDens and InterDens–HighDens edges are displayed in Figure S5 in the Supporting Information. The probability vectors of these pixels have only two nonzero components and the classification uncertainty is limited only to two adjacent classes. From Figures 5 and S5, we observe that set1 leads to a larger number of pixels whose attribution to LowDens or InterDens is uncertain (i.e., located close to *p*_LD_ = *p*_ID_ = 1/2 and *p*_HD_ = 0 in the ternary diagram), whereas set2 leads to a larger number of pixels with uncertain attribution to InterDens or HighDens (i.e., located close to *p*_HD_ = *p*_ID_ = 1/2 and *p*_LD_ = 0 in the ternary diagram).

Considering the average Shannon entropy of pixels attributed to each class (Table 1), the two sets of parameters lead to similar classification uncertainty in most cases (the difference between the average Shannon entropy is within 1 standard deviation). However, set2 yields a less uncertain classification of InterDens for the LowAm and MedAm, whereas set1 leads to a lower uncertainty for the segmentation of the crystalline surfaces.

Note that when set2 is used instead of set1, more pixels lie at the border between HighDens and LowDens, which are noncontiguous in the density scale and result in neighboring HighDens–LowDens pixels which are not separated by InterDens.

In general, the lower classification uncertainty of InterDens with set2 is due to a much larger number of pixels that are almost certainly attributed to InterDens (see Figures 5 and S5). As we expect that the RF leads to a more uncertain classification close to the class boundaries, the lower Shannon entropy of the pixels attributed to InterDens may be due to the smaller interface between InterDens and LowDens which results from the sensibly larger fraction of the surface classified as InterDens (i.e., around 65–67% of the surface area with set2 as against around 50% when set1 is used). Despite the higher Shannon entropy of the InterDens class in the case of amorphous surfaces, the segmentation performed with set1 parameters led to much better defined patterns (Figure 3). As shown below, on average, this leads to better predictions of the CO_2_ residence time and space-averaged density of segmented regions.

Notice that our segmentation algorithm can be applied to any solid/fluid interfaces for which density and/or energy maps characterized by irregular patterns are predicted by numerical simulations^67−69^ and/or measured by spectroscopy techniques.^70−72^ The use of the RF algorithm can be applied to any combination of adsorbates and adsorbents with no a priori restriction on the size of the adsorbate molecules or the physicochemical heterogeneity of the solid substrate, and it is crucial in case the maps display irregular patterns that are not amenable to more standard analysis.

### Segmentation of Crystalline Surfaces and Sorption Dynamics

The segmentation of the crystalline surfaces with the parameters of set1 yields well-defined circularly shaped LowDens regions separated by a connected honeycomb network formed by the InterDens region (Figure 3b). The regularity of the LowDens and InterDens patterns is lost when the segmentation is performed with set2 parameters. Also, set1 yields a total area of 72 nm^2^ for InterDens which is significantly lower than the one attributed by set2, 96 nm^2^ (Table 1).

**Figure 6:**
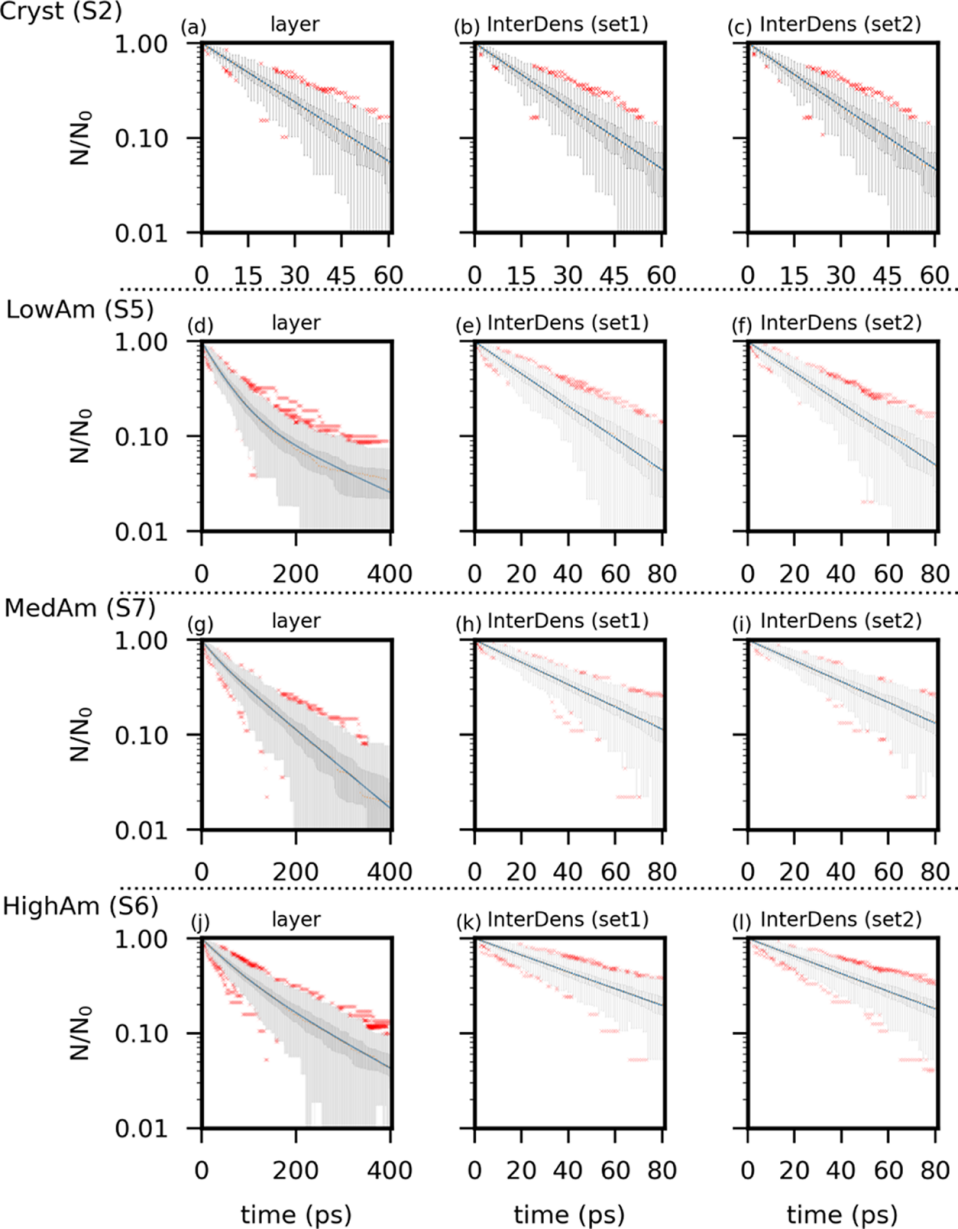
Residence time distribution of CO_2_ molecules, which are adsorbed at the surface layer (a, d, g, j) and within the InterDens regions as segmented by set1 (b, e, h, k) and set2 (c, f, i, l). The residence time is plotted for a representative sample of each surface class (the identification number of the analyzed silica surface is given in brackets, i.e., S2 for Cryst). From top to bottom, the residence-time distribution is shown for crystalline, LowAm, MedAm, and HighAm surfaces. The box plots represent the 0.25–0.75 quartiles (with whiskers extending for 1.5 times the inner quartile range), whereas the red dots are data outliers. The blue solid line is the fitted exponential or double exponential (eq 1).

The marked difference in total area has a negligible impact on the residence time distribution at the InterDens region (see Figure 6b,c for set1 and set2, respectively). Crystalline surfaces are characterized by an exponential distribution of the residence time for both the surface layers and InterDens region. Regardless of the set of parameters used for the segmentation, the mean residence time in the InterDens region is (21 ± 1) ps (Table 2). This indicates that the segmentation performed with set2 overestimates the extension of the InterDens region whose area is 1/3 larger than with set1.

**Table 2:** Time Constant (τ_1_ and τ_2_), Fraction of the Molecules Following τ_1_ (*a*), and Mean Residence Time (τ̅) of Molecules at the Surface Layer and in the InterDens Region Together with the Fraction of Time Spent in Each Class (i.e., LD, ID, and HD) for All 24 Amorphous Surfaces and the 2 Crystalline Surfaces^a^

	decay						adsorption time (%)					
	layer				ID (set1)	ID (set2)	set1			set2		
LowAm	τ_1_ (ps)	τ_2_ (ps)	*a*	τ̅ (ps)	τ (ps)	τ (ps)	LD	ID	HD	LD	ID	HD
S1	53(10)	169(28)	0.50(0.12)	111(26)	37(1)	38(1)	11	40	49	4	39	57
S2	53(8)	163(52)	0.71(0.11)	85(19)	29(1)	31(1)	15	38	47	1	41	58
S3	39(13)	114(13)	0.33(0.13)	89(32)	30(1)	39(1)	17	34	49	2	47	51
S4	24(15)	74(7)	0.22(0.14)	63(37)	36(1)	24(1)	9	56	35	5	38	57
S5	42(4)	190(49)	0.80(0.16)	72(14)	26(1)	27(1)	10	37	53	3	39	58
S6	69(11)	190(55)	0.81(0.21)	92(22)	40(1)	40(1)	9	54	37	4	54	42
S7	37(10)	95(12)	0.34(0.18)	75(35)	38(1)	38(1)	11	47	42	4	47	49
S8	45(10)	123(27)	0.62(0.20)	75(21)	36(1)	28(1)	10	49	41	3	39	58
S1–S8	49(4)	134(11)	0.60(0.07)	83(9)	34(1)	33(1)	12(3)	44(8)	44(6)	3(1)	43(6)	54(6)

MedAm	τ_1_ (ps)	τ_2_ (ps)	*a*	τ̅ (ps)	τ (ps)	τ (ps)	LD	ID	HD	LD	ID	HD
S1	58(7)	232(58)	0.73(0.13)	105(21)	40(1)	40(1)	11	40	49	3	39	58
S2	52(8)	175(41)	0.68(0.12)	91(18)	33(1)	32(1)	12	39	49	3	37	60
S3	56(12)	174(31)	0.49(0.10)	116(25)	41(1)	41(1)	7	40	53	3	39	58
S4	39(7)	97(15)	0.30(0.09)	80(23)	37(1)	32(1)	12	49	39	3	44	53
S5	38(6)	128(21)	0.62(0.15)	72(16)	26(1)	28(1)	8	37	55	3	39	58
S6	43(13)	112(14)	0.33(0.11)	89(27)	39(1)	42(1)	10	48	42	2	51	47
S7	31(12)	104(9)	0.31(0.14)	81(33)	37(1)	39(1)	8	43	49	4	46	50
S8	34(15)	92(12)	0.35(0.12)	72(23)	40(1)	27(1)	9	56	35	4	39	57
S1–S8	48(4)	137(9)	0.52(0.06)	89(9)	37(1)	35(1)	10(2)	44(6)	46(7)	3(1)	42(5)	55(5)

HighAm	τ_1_ (ps)	τ_2_ (ps)	*a*	τ̅ (ps)	τ (ps)	τ (ps)	LD	ID	HD	LD	ID	HD
S1	55(9)	183(39)	0.62(0.08)	104(19)	42(1)	34(1)	9	43	48	3	35	62
S2	47(12)	140(19)	0.39(0.07)	104(20)	37(1)	33(1)	10	39	51	3	36	61
S3	39(14)	112(13)	0.32(0.13)	89(32)	36(1)	35(1)	12	40	48	3	40	57
S4	59(8)	205(55)	0.69(0.12)	104(22)	42(1)	27(1)	8	49	43	3	30	67
S5	42(4)	224(39)	0.71(0.10)	95(15)	25(1)	21(1)	7	30	63	2	26	72
S6	61(17)	161(28)	0.53(0.15)	108(28)	49(1)	47(1)	6	48	46	3	46	51
S7	53(5)	246(8)	0.80(0.09)	92(9)	38(1)	25(1)	7	47	46	3	30	67
S8	54(10)	142(49)	0.74(0.07)	77(16)	32(1)	40(1)	9	43	48	5	53	42
S1–S8	52(3)	168(12)	0.61(0.13)	97(16)	38(1)	37(1)	9(2)	42(6)	49(6)	3(1)	37(9)	60(10)

Cryst	τ_1_ (ps)	τ_2_ (ps)	*a*	τ̅ (ps)	τ (ps)	τ (ps)	LD	ID	HD	LD	ID	HD
S1	22(1)		1		21(1)	21(1)	7	93		5	95	
S2	22(1)		1		21(1)	21(1)	7	93		5	95	

a The uncertainties on the fitted parameters, estimated as the standard deviation obtained from the covariance matrix, are given in parentheses. The rows S1–S8 correspond to the mean behavior of the eight surfaces of each class of amorphous surfaces, i.e., from top to bottom: LowAm, MedAm, and HighAm (the data relative to the eight surfaces in each surface class are fitted together). Note that a single exponential (*a* = 1) is used to fit the crystalline surfaces and the InterDens regions, whereas a double exponential is used in the other cases.

### Segmentation of Amorphous Surfaces and Sorption Dynamics

The density maps of representative amorphous surfaces (one for each class: LowAm, MedAm, and HighAm) are shown in Figure 3, together with the corresponding segmentation obtained with set1 and set2 parameters. The segmentation performed with set1 yields well-defined InterDens regions which are reminiscent of the InterDens network identified on the crystalline surface (Figure 3b). The InterDens region forms a connected network that is distorted by the heterogeneous structure of the amorphous surface, which has a lower surface density of hydroxyl groups than the crystalline surface. InterDens embeds LowDens regions and HighDens regions, which are generated by the interaction between undercoordinated defects and the neighboring hydroxyl groups (see also ref (19)). The total surface areas of LowDens and InterDens are comparable, whereas the HighDens regions are limited in size and number (Table 1). The surface area attributed to each class is displayed for each surface in Figure S6 in the Supporting Information.

The segmentation with set2 yields qualitatively different patterns. The InterDens region covers about 2/3 of the segmented surface (with set1 covering only 1/2 of the surface), and the network structure is lost (for the crystalline surface we observe an analogous change in the segmented patterns when set2 is employed, Figure 3c). The LowDens regions are less homogeneously distributed and considerably smaller, whereas the HighDens regions are present in a larger number and are larger than those obtained with set1.

The different segmentation patterns affect the residence-time distributions of CO_2_ molecules in the InterDens and HighDens regions (see Table 2 and Figure 6).

The residence time in the InterDens region follows an exponential distribution. As for most molecules in the InterDens layer, this is the time elapsed before desorption into the pore; this indicates that the desorption probability of a molecule in the InterDens is constant over time. The values obtained with the two sets of parameters are reported in Table 2 and are comparable, despite the larger area of the InterDens region obtained with set2.

The RF segmentation is able to separate the weakly heterogeneous InterDens network from the highly heterogeneous HighDens regions. Similarly to crystalline surfaces, adsorption in the InterDens region is governed by the interaction between CO_2_ molecules and OH groups, and the residence-time distribution is exponential. In contrast, adsorption in the HighDens regions is dictated by the presence and the specific local arrangement of undercoordinated defects, which are highly heterogeneous. The different strength of interaction between the molecules and the surface in the different segmented objects leads to nonexponential residence-time distribution.

When set2 is employed for the segmentation, molecules spend a larger fraction of time in the HighDens regions (Table 2). This results from the larger surface area attributed to HighDens with respect to the segmentation with set1 (see Table 1). Analogously, the smaller areas attributed to LowDens lead to a smaller fraction of time spent in the LowDens regions (3% instead of 10%).

**Figure 7:**
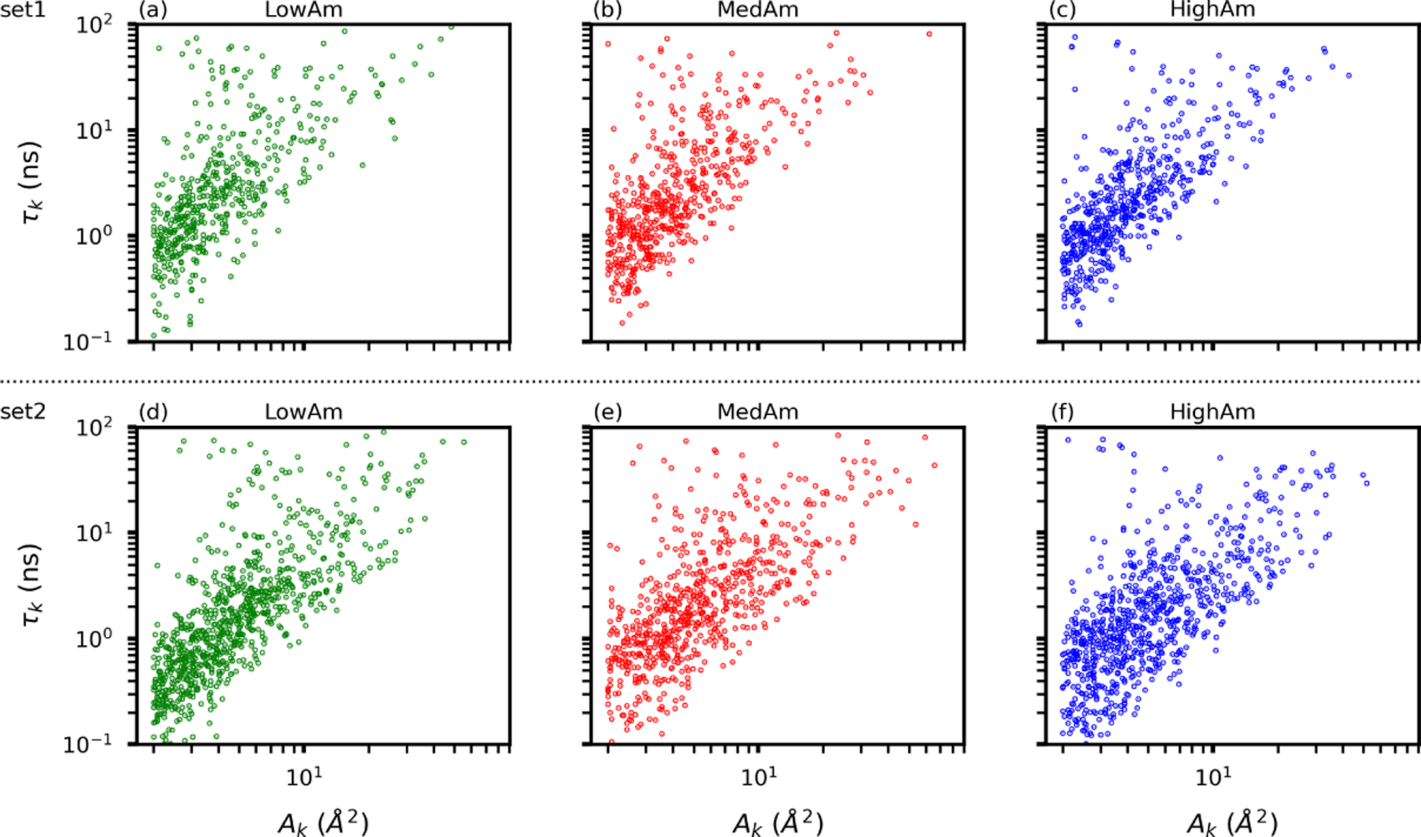
Relationship between the total residence time (τ_*k*_) in the individual HighDens regions and the regions area (*A*_*k*_), for the HighDens regions of all of the surface samples (S1–S8) belonging to LowAm (green), MedAm (red), and HighAm (blue). Panels (a)–(c) refer to set1 predictions, and panels (d)–(f) refer to set2 predictions.

To quantify the heterogeneity of the different segmented objects belonging to the HighDens class (which are characterized by different local combinations and arrangements of undercoordinated defects and hydroxyl groups), for each HighDens object, we plot its area, *A*_*k*_, versus the total residence time, τ_*k*_ (Figure 7). From Figure 7, we can appreciate the heterogeneity of the HighDens objects with values of *A*_*k*_ and τ_*k*_ spanning over 2 and 3 orders of magnitude, respectively. Regardless of the characteristic of the surface (LowAm, MedAm, or HighAm), in the majority of HighDens objects the total residence time of the molecules is in the order of a few nanoseconds. Set2 leads to larger objects, but the distribution of the total residence time is not substantially affected.

**Figure 8:**
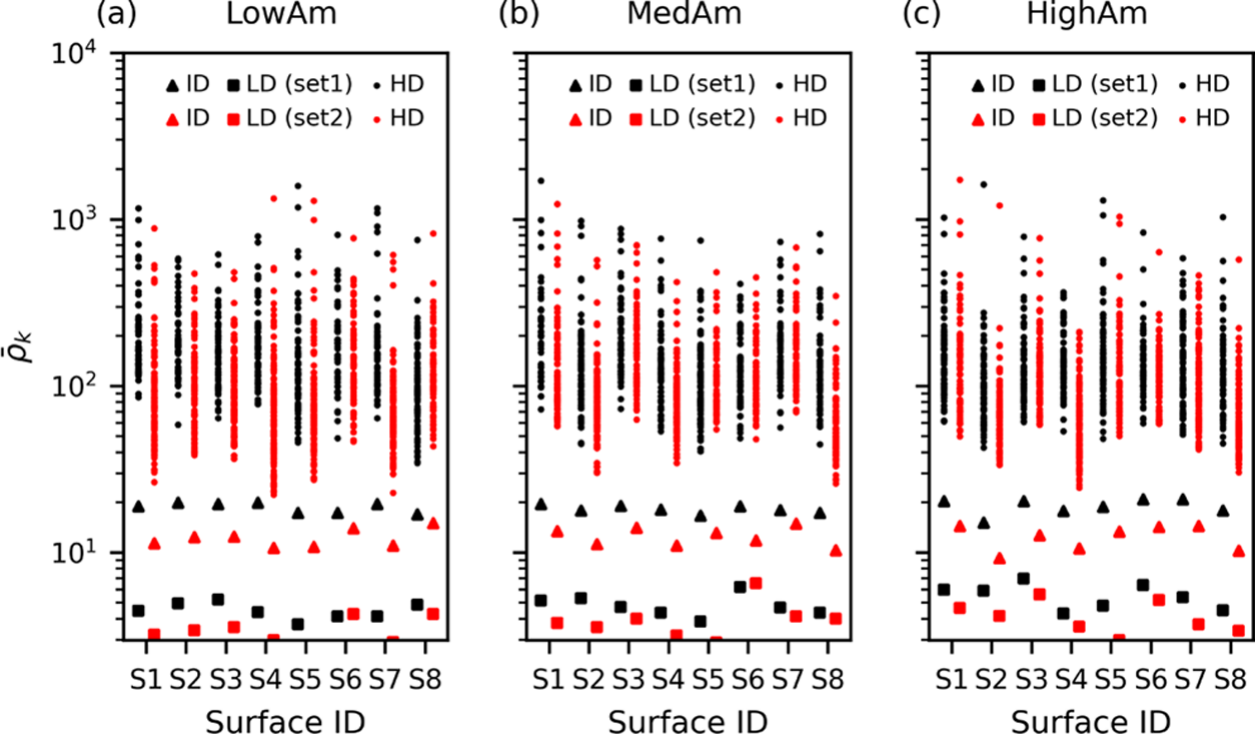
Space-averaged density (ρ̅_*k*_) for each HighDens object and for the LowDens and InterDens classes; LowDens objects are treated as one. Analysis is performed for each of the eight surfaces (S1–S8) of (a) LowAm, (b) MedAm, and (c) HighAm; black and red refer to set1 and set2 predictions, respectively.

Finally, using eq 2, we compute the space-average densities (ρ̅_*k*_) of each HighDens object and we compare it with the ones of the InterDens and LowDens classes (for this analysis LowDens regions are considered as a unique object). From Figure 8, we notice that both sets perform well in separating the three segmentation classes, in terms of their space-averaged densities. On average, set1 yields higher values of ρ̅_*k*_ for the InterDens and LowDens classes. Also for the HighDens class, the object with the lowest value of ρ̅_*k*_ and in general the distributions of all of the HighDens objects appear to be higher in set1 than in set2. Nevertheless, the HighDens classes of set2 are characterized, on average, by 40% more objects than in set1 (see also Figure 3); this makes the overall densities of each map segmented with set1 and set2 (the overall densities being obviously independent from the segmentation set) add up to the same values.

## Conclusions

The interaction of adsorbate molecules with amorphous surfaces is much more complex than that with crystalline surfaces. The topological and chemical heterogeneity of amorphous surfaces leads to a variety of possible interactions, which are characterized by a different bonding strength. To identify surface regions on the basis of their adsorption capacity, we need new tools that are able to segment the surfaces into complex-shaped regions. Here, we propose to use machine learning algorithms to segment the surface into regions that can be then further analyzed and classified to reveal the dynamics of the molecule-surface interaction. Notice that surface segmentation is a prerequisite to allow the calculation of the properties of the subregions (i.e., the residence time in high-density regions, which differs markedly from that of the layer, cannot be computed from the MD simulations without first partitioning the surfaces into appropriate subregions of irregular shape).

We use an RF classifier to segment the surface density maps (i.e., the map of the spatial distribution of the time-averaged density of the molecules adsorbed on the surface), which typically display a connected region characterized by intermediate density, disseminated by regions with a sensibly higher density, and bounded by lower-density regions (in which almost no adsorption takes place). RF classifies the pixels of the density map based on multiple features (intensity, gradient, and the two eigenvalues of the Hessian matrix obtained by applying different filtering levels) which allows a nonlocal segmentation, which takes into account not only the characteristic of individual pixels, but also those of their neighbors. This allows preservation of local structures that would not be possible, for instance, by simple thresholding, which segments the maps based only on individual pixel intensities.

The HighDens (respectively, LowDens) regions are naturally found around maxima (respectively, minima) of the density maps, whereas InterDens regions are close to saddle points, as they typically bridge higher-density regions and are bound by lower-density regions. This gives us a criterion to define the subregions from which we select the labeled data to train the RF classifier. By modifying the boundary between these regions, we can control pixel classification and obtain considerably different segmentation patterns. Several criteria can be used to evaluate the quality of the segmentation and to discriminate among different sets of segmentation parameters. For instance, parameters that minimize segmentation uncertainty (quantified, e.g., by Shannon entropy) can be preferred. Ultimately, however, the parameters should be selected to serve the specific purpose of the segmentation and the prediction of the related target properties.

Here, we consider an example in which we aim at separating highly heterogeneous regions (HighDens) from the weakly heterogeneous regions (InterDens), which exhibit simpler adsorption dynamics and an elementary residence-time distribution.

We demonstrate that the RF segmentation can be effectively used to identify a weakly heterogeneous InterDens region, which is characterized by an exponential distribution of the residence time and has a behavior similar to the crystalline surface (which also exhibits an exponential residence-time distribution). In contrast, the segmented HighDens regions are very heterogeneous, with a complex residence-time distribution resulting from the presence of undercoordinated defects. On each surface, the HighDens class consists of several disconnected objects with total residence times and areas varying greatly from one object to the other (typically, 3 and 2 orders of magnitude for the time and the area, respectively). This heterogeneity is responsible for the complex residence time distribution of the HighDens regions and, ultimately, of the entire amorphous surface layer (which cannot be described by a simple exponential^19^).

Once the different adsorption regions are identified, they can be further characterized and classified. We envisage that the segmented regions can be correlated to the underlying atomic disposition of the atoms on the amorphous surfaces. For instance, once the HighDens regions are obtained from RF segmentation, ML algorithms can be used to relate the characteristics of different classes of HighDens objects to specific features of the underlying arrangements of the undercoordinated defects, such as Si3 and NBO, and hydroxyl groups. This can reveal surface features that can be designed to increase the CO_2_ adsorption capacity or even to participate in catalyzing reactions of broad interest such as CO_2_ methanation^2,3^ and methane reforming.^73^ Flexible segmentation algorithms are also indispensable to enable calculation of transport, adsorption, and reaction properties that can be used in upscaled models of amorphous materials.

For instance, a kinetic Monte Carlo (KMC) model can be developed based on the properties computed for the different adsorption regions (i.e., residence time constants). A reliable KMC model would enable the modeling of the adsorption dynamic onto much larger surfaces and for a much longer time than what is possible with MD, allowing us to greatly reduce computational cost and to obtain meso- and large-scale quantities that can be more conveniently compared with experimental observations.

## Supporting Information Available

CO_2_ density profiles, fraction of surface sites, pixels uncertainty analysis, and area attributed to each class (PDF)
