# Supplementary material for: Learning Adsorption Patterns on Amorphous Surfaces
Source: J Chem Theory Comput. 2024 Aug 26;20(17):7597–610. doi: 10.1021/acs.jctc.4c00702 (PMC11391580; doi:10.1021/acs.jctc.4c00702)
Supplement: Supplementary file 1 — ct4c00702_si_001.pdf [file ct4c00702_si_001.pdf]

Supplementary Information for: Learning adsorption patterns on amorphous surfaces

Mattia Turchi,*,† Sandra Galmarini,‡ and Ivan Lunati*,†

†*Laboratory for Computational Engineering, Swiss Federal Laboratories for Materials Science and Technology, Empa, Überlandstrasse 129, 8600 Dübendorf, Switzerland*

‡*Laboratory for Building Energy Materials and Components, Swiss Federal Laboratories for Materials Science and Technology, Empa, Überlandstrasse 129, 8600 Dübendorf, Switzerland*

E-mail: mattia.turchi@empa.ch; ivan.lunati@empa.ch

Fraction of different groups at amorphous surfaces

Table S1: Fraction of different groups at the surface.

LowAm	OH/nm^2	Si3/nm^2	NBO/nm^2
S_1	1.8	0.24	0.3
S_2	1.5	0.19	0.2
S_3	1.6	0.21	0.2
S_4	2.0	0.10	0.1
S_5	1.7	0.25	0.2
S_6	1.6	0.12	0.2
S_7	2.0	0.23	0.2
S_8	1.7	0.16	0.1
MedAm	OH/nm^2	Si3/nm^2	NBO/nm^2
S_1	2.2	0.25	0.27
S_2	2.2	0.20	0.17
S_3	2.2	0.26	0.32
S_4	2.2	0.24	0.26
S_5	2.4	0.29	0.24
S_6	2.3	0.17	0.29
S_7	2.2	0.29	0.18
S_8	2.1	0.26	0.28
HighAm	OH/nm^2	Si3/nm^2	NBO/nm^2
S_1	2.8	0.32	0.21
S_2	2.9	0.26	0.46
S_3	2.7	0.25	0.37
S_4	3.0	0.35	0.24
S_5	2.8	0.29	0.22
S_6	2.9	0.27	0.35
S_7	2.7	0.19	0.18
S_8	2.8	0.24	0.23

Schematic representation of the MD-simulated system

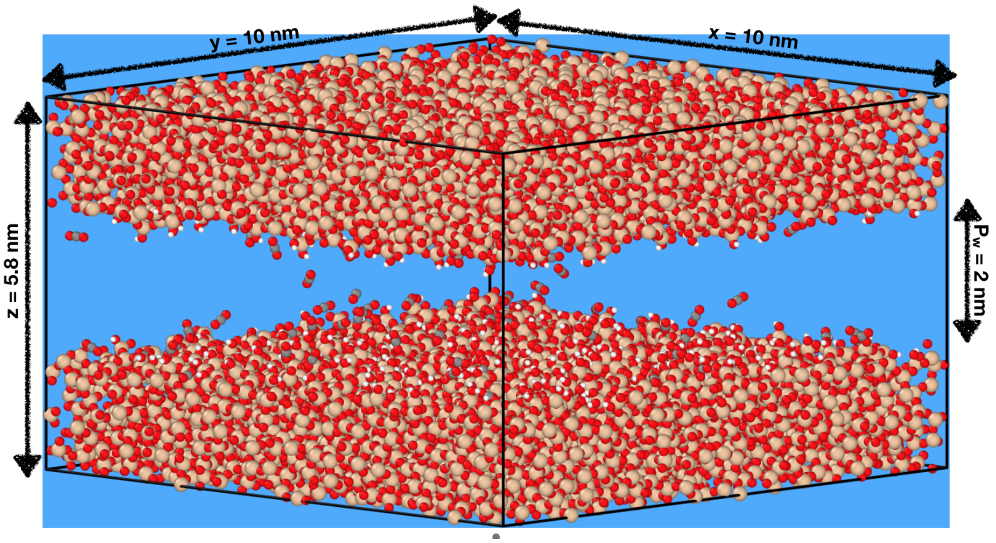

Figure S1: Representation of the amorphous silica pore populated with 100 CO_2 molecules, the fully periodic simulation box extends for 10 nm in x and y and for 5.8 nm in z directions. The pore width is of 2 nm. Atoms have the following color-code: Oxygen (red), carbon (gray), silicon (gold) and hydrogen (white).

Definition of surface layer

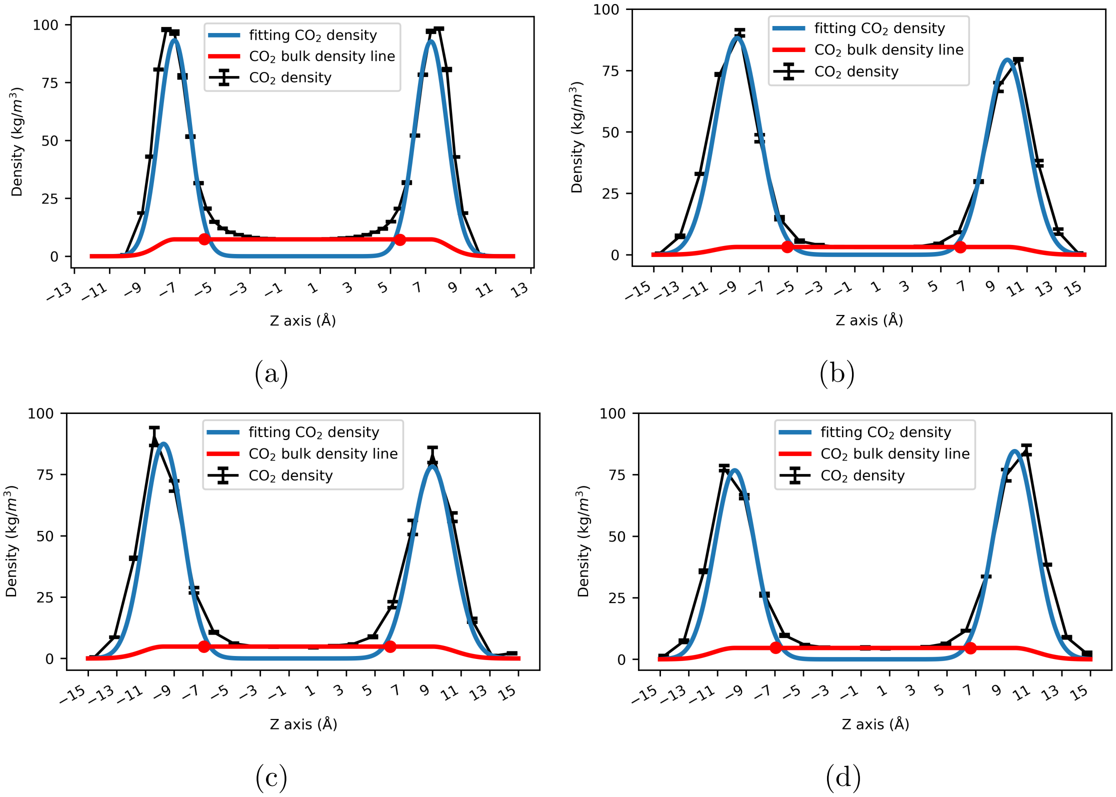

Figure S2: Density profiles for CO_2 at the amorphous pores surfaces: a) Fully hydroxylated crystal pore (FullCr) b) High hydroxylation amorphous pore (HighAm), c) Medium hydroxylation amorphous pore (MedAm), d) Low hydroxylation amorphous pore (LowAm).

In each of the four panels of Figure S2, we see the two peaks relative to the excess of CO_2 density close to the pore surfaces, indicating the presence of adsorbed CO_2, and a much lower CO_2 density at the center of the pore from non-adsorbed molecules. To calculate the cutoff distance that defines the interface layer, we first determine the constant background density of CO_2 at the center of the pore; then we fit the two peaks of CO_2 excess to two independent Gaussian functions. There is some ambiguity in defining the exact limit of the layer characterized by a density excess. To exclude molecules that are weakly bounded, we define the cutoff as the distance from the wall at which the CO_2 density is twice the bulk density. We assume that this estimates the distance at which a randomly chosen CO_2 molecule is more likely to be desorbed than adsorbed. Therefore, the layer cutoffs are defined

as the points of intersection between the two Gaussian and the constant background density. A CO_2 molecule is considered to be adsorbed if the value of the z coordinate is within the cutoff values.

Pre-processing of the surface density maps

The adsorption density maps are generated by mapping the x,y coordinates of the carbon atoms of adsorbed CO_2 molecules onto a 2D histogram of 500x500 bins, a total of 90,000 frames over the last 90ns are considered. Due to the presence of the high density regions, which display density values up to 100 times more than the intermediate density regions, the resulting density map is characterized by low contrast, Figure S3a, therefore a logarithmic transformation is operated to increase the visibility of the intermediate density regions Figure S3b. The resulting density maps still presents background noise which is removed by means of the total variation algorithm as presented by Chambolle et al.,[1] Figure S3c and by applying a Gaussian smoothing with a standard deviation for the kernel of $\sigma = 1$.

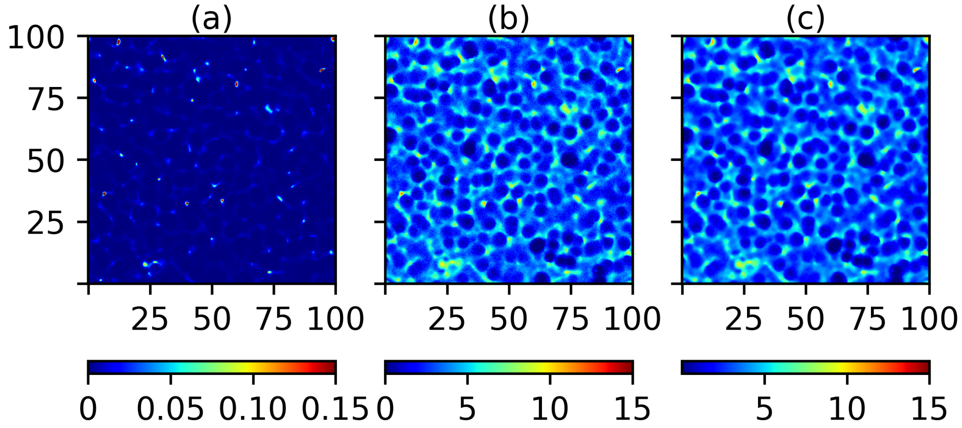

Figure S3: CO_2 density maps: low contrast image (a), contrast enhancement by logarithmic transformation (b).

Shannon entropy ($\tilde{H}$) for different segmentation

Table S2: Median value of the Shannon entropy metric ($\tilde{H}$) for the 16 different segmentation performed varying the values of $-K_{\mathrm{E2,ID}}$ and $-K_{\mathrm{E2,HD}}$ by means of a grid search. The set of constants yielding the lowest value of $\tilde{H}$ (which are selected to compute the target properties) for the two types of segmentation are highlighted in the table, set1 (blue) and set2 (red).

LowAm																
$-K_{\mathrm{E2,ID}}$	0.2	0.2	0.2	0.2	0.15	0.15	0.15	0.15	0.1	0.1	0.1	0.1	0	0	0	0
$-K_{\mathrm{E2,HD}}$	0.2	0.15	0.1	0	0.2	0.15	0.1	0	0.2	0.15	0.1	0	0.2	0.15	0.1	0
S1	0.69	0.70	0.69	0.68	0.67	0.65	0.68	0.66	0.69	0.64	0.69	0.65	0.44	0.46	0.26	0.25
S2	0.67	0.69	0.68	0.68	0.62	0.63	0.63	0.66	0.62	0.63	0.61	0.58	0.58	0.57	0.55	0.53
S3	0.67	0.67	0.68	0.67	0.64	0.63	0.62	0.64	0.68	0.68	0.66	0.66	0.40	0.44	0.41	0.44
S4	0.66	0.68	0.68	0.65	0.69	0.67	0.67	0.66	0.65	0.66	0.62	0.64	0.54	0.48	0.52	0.51
S5	0.69	0.70	0.69	0.69	0.65	0.69	0.66	0.66	0.58	0.57	0.58	0.60	0.59	0.59	0.32	0.33
S6	0.67	0.68	0.70	0.69	0.69	0.69	0.69	0.70	0.64	0.67	0.68	0.65	0.46	0.47	0.43	0.40
S7	0.67	0.68	0.66	0.65	0.65	0.66	0.67	0.63	0.68	0.68	0.63	0.61	0.57	0.51	0.49	0.48
S8	0.66	0.67	0.69	0.65	0.61	0.64	0.62	0.69	0.66	0.68	0.63	0.64	0.47	0.47	0.49	0.47
MedAm																
$-K_{\mathrm{E2,ID}}$	0.2	0.2	0.2	0.2	0.15	0.15	0.15	0.15	0.1	0.1	0.1	0.1	0	0	0	0
$-K_{\mathrm{E2,HD}}$	0.2	0.15	0.1	0	0.2	0.15	0.1	0	0.2	0.15	0.1	0	0.2	0.15	0.1	0
S1	0.68	0.69	0.70	0.69	0.67	0.67	0.68	0.68	0.68	0.66	0.67	0.67	0.46	0.45	0.49	0.31
S2	0.69	0.70	0.70	0.70	0.69	0.68	0.66	0.64	0.66	0.67	0.62	0.65	0.49	0.52	0.45	0.46
S3	0.66	0.70	0.69	0.70	0.65	0.67	0.67	0.68	0.62	0.65	0.66	0.60	0.38	0.26	0.24	0.26
S4	0.62	0.63	0.64	0.64	0.62	0.65	0.61	0.66	0.59	0.63	0.64	0.65	0.42	0.41	0.46	0.44
S5	0.67	0.66	0.64	0.66	0.64	0.63	0.62	0.63	0.63	0.63	0.66	0.61	0.32	0.29	0.30	0.21
S6	0.65	0.67	0.65	0.66	0.66	0.64	0.64	0.62	0.58	0.63	0.66	0.63	0.52	0.53	0.53	0.53
S7	0.68	0.58	0.67	0.68	0.62	0.61	0.62	0.64	0.63	0.61	0.63	0.63	0.41	0.40	0.27	0.21
S8	0.67	0.63	0.65	0.67	0.69	0.66	0.68	0.68	0.68	0.68	0.66	0.68	0.50	0.46	0.50	0.53
HighAm																
$-K_{\mathrm{E2,ID}}$	0.2	0.2	0.2	0.2	0.15	0.15	0.15	0.15	0.1	0.1	0.1	0.1	0	0	0	0
$-K_{\mathrm{E2,HD}}$	0.2	0.15	0.1	0	0.2	0.15	0.1	0	0.2	0.15	0.1	0	0.2	0.15	0.1	0
S1	0.67	0.68	0.68	0.62	0.67	0.66	0.69	0.68	0.63	0.67	0.61	0.60	0.57	0.50	0.45	0.37
S2	0.69	0.66	0.68	0.67	0.65	0.65	0.56	0.61	0.60	0.59	0.64	0.63	0.50	0.52	0.54	0.54
S3	0.68	0.70	0.71	0.66	0.64	0.65	0.65	0.62	0.65	0.65	0.66	0.59	0.45	0.48	0.46	0.49
S4	0.61	0.62	0.65	0.62	0.63	0.64	0.60	0.63	0.61	0.59	0.57	0.58	0.54	0.57	0.54	0.54
S5	0.69	0.66	0.62	0.63	0.65	0.63	0.63	0.62	0.66	0.67	0.63	0.61	0.45	0.47	0.49	0.50
S6	0.62	0.67	0.63	0.67	0.69	0.66	0.68	0.64	0.61	0.65	0.57	0.55	0.47	0.45	0.42	0.38
S7	0.67	0.68	0.68	0.68	0.68	0.67	0.69	0.68	0.65	0.67	0.66	0.67	0.60	0.59	0.57	0.60
S8	0.66	0.65	0.70	0.66	0.62	0.65	0.63	0.66	0.67	0.63	0.66	0.65	0.50	0.42	0.26	0.38

Examples of the 16 different segmentation for S1 of HighAm

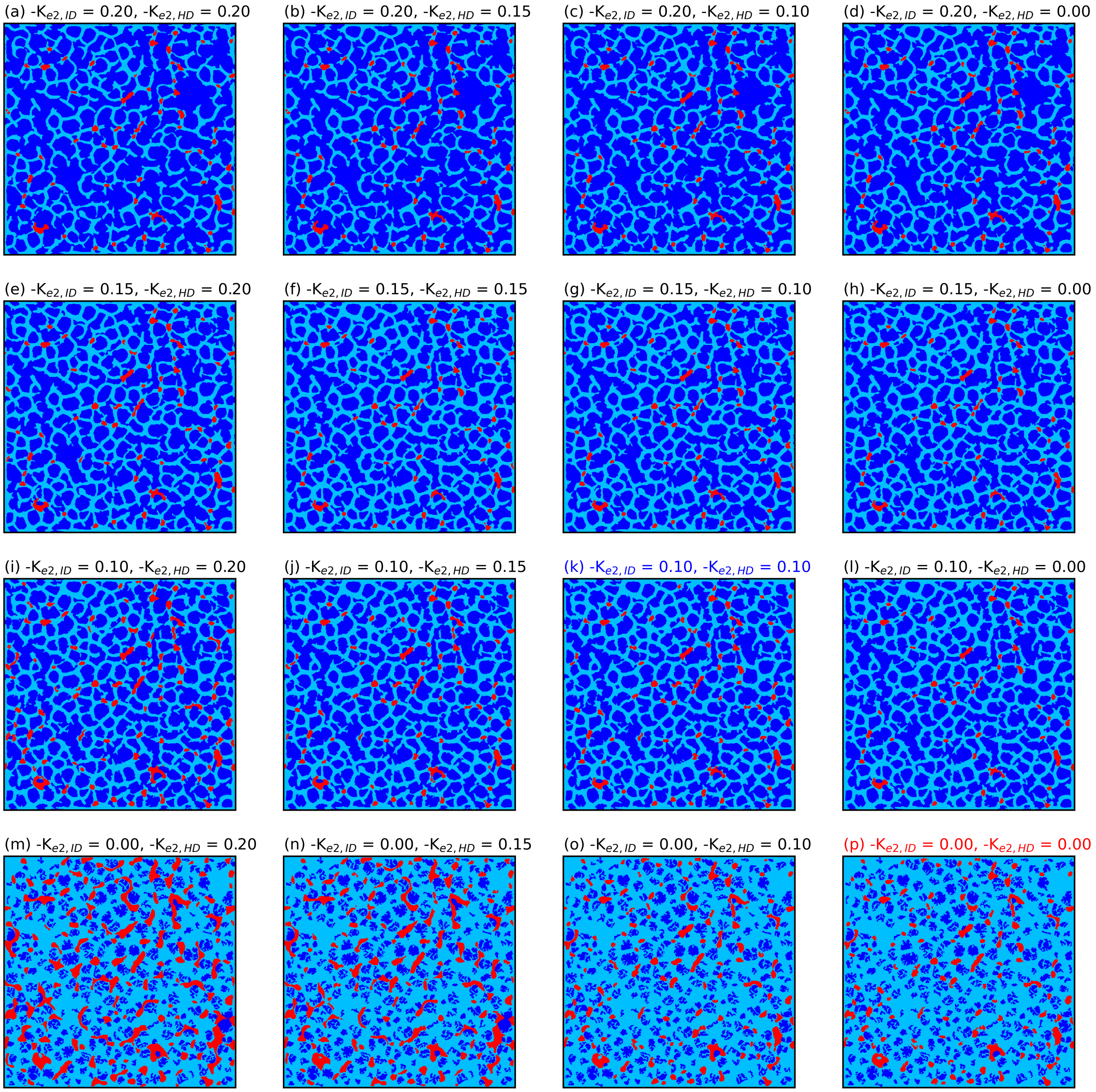

Figure S4: Example of the 16 different segmented CO_2 density maps obtained with all the considered combination of -$K_{\mathrm{E2,ID}}$ and -$K_{\mathrm{E2,HD}}$. Segmented regions for the LowDens, InterDens and HighDens classes are colored in blue, light blue and red respectively. The segmentation characterized by the lowest $\tilde{H}$ (which are selected to compute the target properties) are the ones of panel (k) for set1 and panel (p) for set2.

Areas and $\tilde{H}$ factors for each segmentation class and pores surface

Table S3: Area and median values of H factors ($\tilde{H}$) attributed to each class (LowDens, MedDens, HighDens) for the two set of parameters with different values of E^1 and -$K_{E2,ID}$ and -$K_{E2,HD}$ as selected in Table S2.

Low Am	LowDens (A [nm^2], $\tilde{H}$)		InterDens (A [nm^2], $\tilde{H}$)		HighDens (A [nm^2], $\tilde{H}$)	
	A1, A2	H1, H2	A1, A2	H1, H2	A1, A2	H1, H2
S1	47, 26	0.67, 0.69	48, 68	0.50, 0.19	5, 6	0.55, 0.55
S2	44, 22	0.58, 0.64	53, 69	0.54, 0.47	3, 9	0.58, 0.61
S3	57, 22	0.63, 0.64	39, 72	0.58, 0.27	4, 6	0.56, 0.55
S4	41, 26	0.64, 0.63	56, 63	0.62, 0.41	3, 11	0.55, 0.56
S5	53, 28	0.61, 0.61	44, 66	0.52, 0.24	3, 6	0.54, 0.59
S6	51, 25	0.65, 0.67	45, 70	0.56, 0.17	4, 5	0.54, 0.50
S7	52, 26	0.66, 0.67	45, 67	0.54, 0.46	3, 7	0.56, 0.65
S8	43, 25	0.65, 0.67	54, 64	0.63, 0.34	3, 11	0.52, 0.58
Med Am	LowDens (A [nm^2], $\tilde{H}$)		InterDens (A [nm^2], $\tilde{H}$)		HighDens (A [nm^2], $\tilde{H}$)	
	A1, A2	H1, H2	A1, A2	H1, H2	A1, A2	H1, H2
S1	51, 26	0.69, 0.69	46, 69	0.57, 0.21	3, 5	0.53, 0.52
S2	46, 26	0.65, 0.65	50, 65	0.53, 0.39	4, 9	0.56, 0.61
S3	45, 27	0.66, 0.67	51, 68	0.60, 0.13	4, 5	0.24, 0.53
S4	50, 25	0.69, 0.69	46, 68	0.69, 0.31	4, 7	0.50, 0.56
S5	46, 29	0.64, 0.66	50, 65	0.59, 0.15	4, 6	0.58, 0.53
S6	52, 23	0.58, 0.61	43, 69	0.58, 0.40	5, 8	0.63, 0.61
S7	39, 27	0.66, 0.63	57, 69	0.49, 0.06	4, 4	0.57, 0.53
S8	46, 25	0.67, 0.68	50, 65	0.63, 0.42	4, 10	0.57, 0.56
High Am	LowDens (A [nm^2], $\tilde{H}$)		InterDens (A [nm^2], $\tilde{H}$)		HighDens (A [nm^2], $\tilde{H}$)	
	A1, A2	H1, H2	A1, A2	H1, H2	A1, A2	H1, H2
S1	48, 27	0.61, 0.66	49, 65	0.48, 0.25	3, 8	0.06, 0.58
S2	51, 28	0.59, 0.64	45, 65	0.11, 0.49	4, 7	0.16, 0.62
S3	46, 27	0.64, 0.69	50, 66	0.47, 0.48	4, 7	0.53, 0.50
S4	54, 28	0.59, 0.65	43, 63	0.52, 0.48	3, 9	0.37, 0.65
S5	52, 29	0.63, 0.62	44, 62	0.55, 0.44	4, 9	0.56, 0.60
S6	38, 27	0.69, 0.66	59, 69	0.56, 0.14	3, 4	0.49, 0.54
S7	43, 25	0.69, 0.69	54, 61	0.46, 0.52	3, 14	0.66, 0.63
S8	48, 27	0.63, 0.59	49, 68	0.63, 0.16	3, 5	0.58, 0.56
Cryst	LowDens (A [nm^2], $\tilde{H}$)		InterDens (A [nm^2], $\tilde{H}$)		HighDens (A [nm^2], $\tilde{H}$)	
	A1, A2	H1, H2	A1, A2	H1, H2	A1, A2	H1, H2
S1	28, 5	0.63, 0.69	72, 95	0.02, 0.05	-, -	-, -
S2	28, 4	0.63, 0.69	72, 96	0.02, 0.07	-, -	-, -

N° of pixels in bins at the LD-ID and ID-HD edges of ternary plot

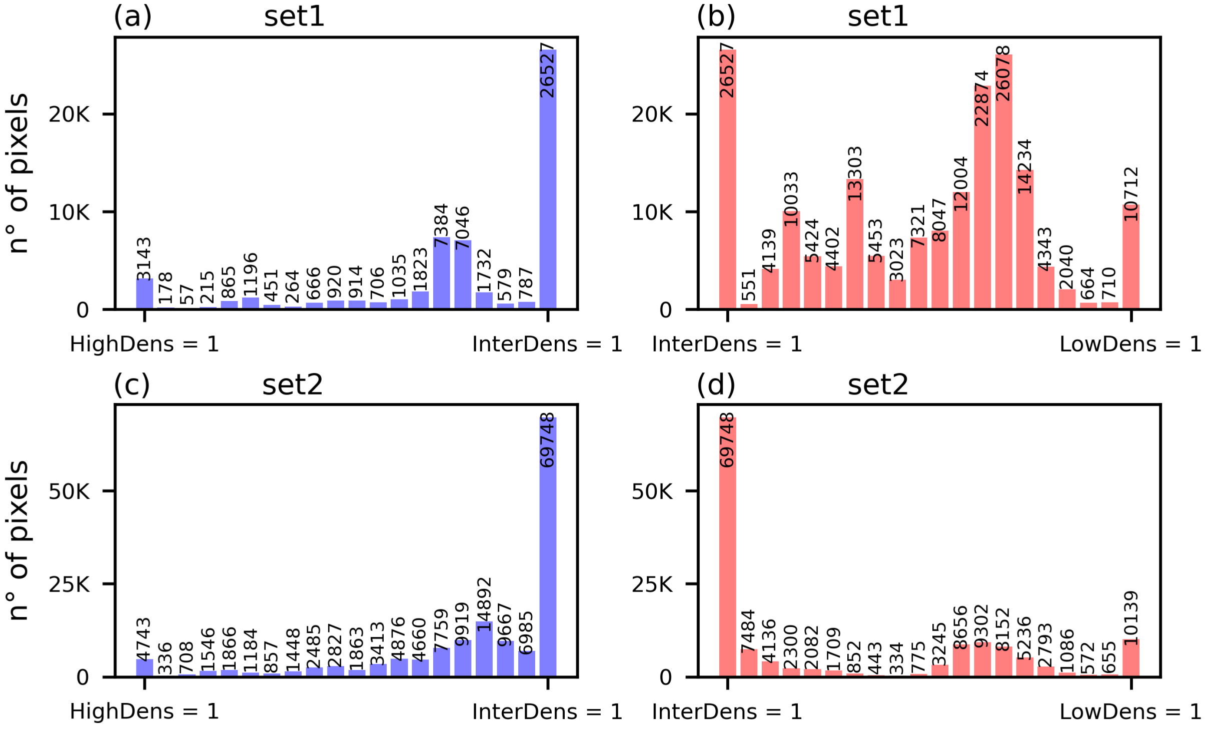

Figure S5: Number of pixels (each bar is labelled with the exact number) in the bins at the LowDens-InterDens and InterDens-HighDens edges of the ternary diagram for set1 (a,b) and set2 (c,d).

Areas for each segmentation class and pores surface

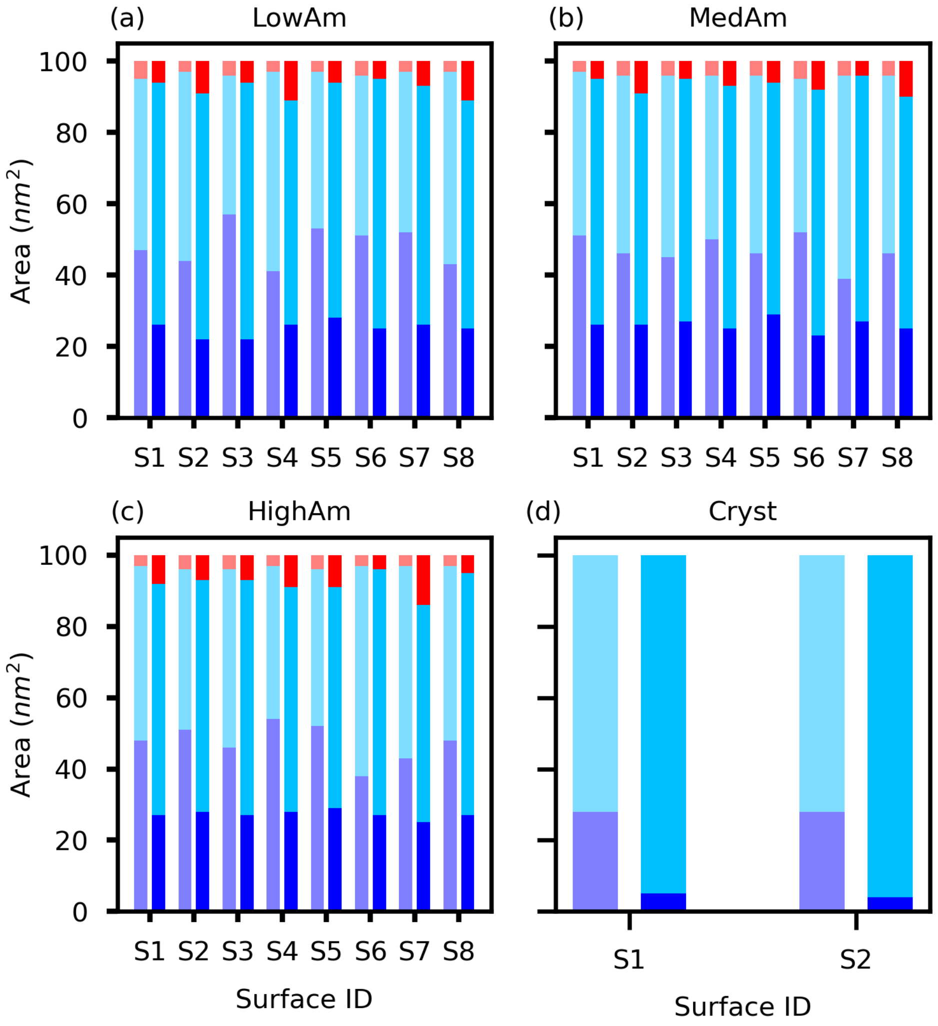

Figure S6: Bars chart of the cumulative areas (for all the surfaces, S1-S8 for amorphous pores and S1-S2 the for crystalline pore) attributed to each class: LowDens (dark blue), MedDens (light blue), HighDens (red) for a) LowAm, b) MedAm, c) HighAm and d) Cryst pores classes. Bars relative to segmentation performed with set1 (left bar for each surface) and set2 (right bar for each surface) are displayed with opaque and no-opaque colors, respectively.

References

(1) Chambolle, A. An algorithm for total variation minimization and applications. *Journal of Mathematical imaging and vision* **2004**, *20*, 89–97.
